# Extracellular vesicles and their RNA cargo facilitate bidirectional cross-kingdom communication between human and bacterial cells

**DOI:** 10.1080/19490976.2026.2630482

**Published:** 2026-02-20

**Authors:** Laura Gröger, Shusruto Rishik, Nicole Ludwig, Amila Beganovic, Marcus Koch, Stefanie Rheinheimer, Martin Hart, Petra König, Tabea Trampert, Pascal Paul, Annette Boese, Claus-Michael Lehr, Sören L. Becker, Gregor Fuhrmann, Andreas Keller, Eckart Meese

**Affiliations:** a Saarland University (USAAR), Chair for Clinical Bioinformatics, Center for Bioinformatics, Saarbrücken, Germany; b Saarland University (USAAR), NGS Sequencing Facility, Homburg, Germany; c INM - Leibniz Institute for New Materials, Saarbrücken, Germany; d Saarland University of Applied Sciences, Institute for Physical Process Technology, Saarbrücken, Germany; e Saarland University (USAAR), Department of Human Genetics, Homburg, Germany; f Saarland University (USAAR), Center for Human and Molecular Biology (ZHMB), Homburg, Germany; g Helmholtz Institute for Pharmaceutical Research Saarland (HIPS), Saarbrücken, Germany; h Saarland University (USAAR), Department of Pharmacy, Saarbrücken, Germany; i Saarland University (USAAR), Institute of Medical Microbiology and Hygiene, Homburg, Germany; j Friedrich-Alexander-University (FAU) Erlangen-Nürnberg, Department of Biology, Pharmaceutical Biology, Erlangen, Germany; k Saarland University (USAAR), PharmaScienceHub (PSH), Saarbrücken, Germany

**Keywords:** Extracellular vesicles, EV, communication, cross-kingdom, bacteria, miRNA

## Abstract

While extracellular vesicles (EVs) are established mediators of intra-species signaling, their contribution to cross-kingdom communication remains incompletely understood. Here, we investigate the EV-mediated interactions between human colon epithelial cells and both Gram-positive and Gram-negative gut bacteria. We show that bacterial EVs (BEVs) derived from *Lacticaseibacillus casei*, *Enterococcus faecalis*, and *Proteus mirabilis* induce distinct transcriptomic changes in Caco-2 cells depending on the bacterial species, with up to ~6,000 differentially expressed genes, including *CCL20*, *CXCL8*, or *CXCL10*. Transfection of BEV-derived RNA independently induces a subset of similar effects, indicating that the EV-mediated communication is partially driven by the RNA cargo. Conversely, we demonstrate that bacteria interact with Caco-2-derived EVs and miR-192-5p, which is highly abundant (~36.4-fold higher) in EVs isolated from conditioned medium compared with EVs from unconditioned medium, with modest effects on bacterial growth. Furthermore, we show that lipid-based packaging of miR-192-5p modulates its association with the bacteria. Our findings support a conceptual model in which EVs and their RNA cargo contribute to species-dependent host-microbe interactions. This study introduces a framework for understanding EVs as cross-kingdom regulators and underscores the importance of tailored, context-specific analyses for understanding the scope of EV-mediated interactions in microbiome-host homeostasis and disease.

## Article highlights

- *L. casei*, *E. faecalis,* and *P. mirabilis* produce BEVs, which accumulate within Caco-2 cells upon incubation. *L. casei*-derived BEVs positively affect the viability of Caco-2 cells. Incubation of Caco-2 cells with BEVs leads to changes in the expression of immune response-related genes such as *CCL20*, *CXCL8,* or *CXCL10*.
- BEVs carry RNAs, and the RNA cargo varies between BEVs from the different bacterial species. Comparison of Caco-2 gene deregulation following incubation with BEVs or transfection with BEV-RNA highlights component-specific effects.
- *E. faecalis* shows modestly altered growth in the presence of Caco-2 EVs. MiRNA-192-5p is frequently detected in EVs from Caco-2 cells. *P. mirabilis* interacts with synthetic miR-192-5p, and the interaction of *L. casei* and *E. faecalis* with this miRNA can be altered by lipid-based packaging of the miRNA.

## Introduction

Communication between cells is essential for building and maintaining complex organization in higher organisms. While this communication can occur through direct cell-to-cell contact or by secretion of signaling molecules into extracellular fluids to reach distant recipient cells,^1^ researchers have proposed that extracellular vesicles (EVs) act as versatile and conserved mediators of cell-to-cell communication.^2^ EVs are small membrane-bound particles naturally released into the extracellular space. They are produced by most eukaryotes and prokaryotes and transport various molecules like proteins, lipids, and nucleic acids.^3^ Among various classes of RNAs, mammalian EVs, such as exosomes and microvesicles, are frequently observed to carry microRNAs (miRNAs),^4-6^ which are known to act as important posttranscriptional regulators of gene expression.^7^ Similarly, bacterial EVs (BEVs), such as membrane vesicles (MVs) produced by Gram-positive bacteria or outer membrane vesicles (OMVs) derived from Gram-negative bacteria, have been found to carry different classes of RNAs, although the composition and abundance of RNA cargo can vary significantly depending on the bacterial species and the vesicle biogenesis pathway.^8-11^

While EVs are well established as participants in intra-species communication, more recent evidence suggests that they also actively facilitate cross-kingdom interactions.^2^ Various studies indicate that BEVs can be transferred to eukaryotic cells and modulate host gene expression,^12-14^ with bacterial RNA cargo playing a significant role in the modulation of the host cellular response.^11^^,^^15^^,^^16^ Despite broad agreement on the relevance of BEVs in bacterial-mammalian cross-kingdom communication, key mechanistic aspects remain debated, and the extent to which BEV-associated RNAs contribute to gene regulation in mammalian cells remains incompletely understood. This gap in knowledge is especially relevant given the association of BEVs with human diseases such as inflammatory bowel disease.^17^ It is therefore crucial to gain deeper insights into these communication processes at the highest possible resolution. Furthermore, even in diseases not directly associated with the gut—such as Parkinson’s disease (PD)—changes in the gut microbiome composition are frequently observed,^18^^,^^19^ raising the possibility that BEVs may contribute to disease development and progression. Conversely, several studies showed that human and murine miRNAs can be taken up by bacterial cells and can affect their growth and gene expression.^20-24^ However, the impact of host-derived EVs and their RNA cargo on gut bacteria remains similarly underexplored.

Here, we address this gap by proposing that EVs and their RNA cargo mediate a bidirectional, species-specific molecular dialog, using a model system comprising human colon epithelial cells as well as Gram-positive and Gram-negative gut bacteria. We selected different members of the order Lactobacillales and Enterobacterales, which have been frequently associated with dysbiosis in PD.^25^ *Lacticaseibacillus casei* (*L. casei*) has been associated with anti-inflammatory properties and specific strains are used as probiotics to improve gastrointestinal symptoms in PD patients.^26-28^ *Enterococcus faecalis* (*E. faecalis*) has gained attention due to its ability to metabolize the PD medication levodopa (L-dopa) using bacterial tyrosine decarboxylase, thereby potentially influencing therapeutic efficacy.^29^^,^^30^ Furthermore, oral administration of *Proteus mirabilis* (*P. mirabilis*) isolated from a murine PD model has been observed to cause damage of dopaminergic neurons and stimulate *α*-synuclein aggregation in the colon and brain of mice.^31^ Collectively, these bacterial species cover diverse mechanisms by which they influence PD and can directly interact with the intestinal epithelium. Despite this connection, it remains largely underexplored whether BEVs derived from *L. casei*, *E. faecalis* and *P. mirabilis* and their RNA cargo contribute to the communication between these bacteria and gut epithelial cells.

Here, we systematically dissect the interaction of Caco-2 cells with BEVs from *L. casei*, *E. faecalis*, and *P. mirabilis* as well as the effects of BEVs and BEV-derived RNA on host cell viability and the transcriptome. Conversely, we analyze the miRNA expression profile of Caco-2-derived EVs and explore whether bacteria are able to interact with host-derived EVs and EV-miRNAs. This work reframes EVs as active participants in host-microbiome regulation with potential implications for health and disease.

## Materials and methods

### Bacterial cultures

*L. casei* (DSM 20011) was grown in deMan, Rogosa and Sharpe (MRS) broth (Carl Roth, Karlsruhe, Germany), *E. faecalis* (DSM 20478), and *P. mirabilis* (DSM 4479) were grown in brain heart infusion (BHI) broth (Merck Millipore, Burlington, MA, USA). Bacterial growth was monitored by measuring the optical density (OD) at a wavelength of *λ* = 600 nm or by determining the colony forming units per milliliter (CFU/ml). Liquid cultures were grown statically at 37 °C under ambient atmospheric conditions, starting from an OD of 0.1 until reaching the stationary phase of growth (Supplementary Figure 1A–C).

### Eukaryotic cell culture

Human colon adenocarcinoma cells, Caco-2 (ATCC HTB-37), were maintained in Dulbecco‘s Modified Eagle‘s Medium (DMEM, Thermo Fisher Scientific, Waltham, MA, USA) supplemented with 10% (v/v) fetal calf serum (FCS, Thermo Fisher Scientific, Waltham, MA, USA) and 1% (v/v) non-essential amino acids (NEAA, Thermo Fisher Scientific, Waltham, MA, USA) at 37 °C and 5% CO_2_. Medium was exchanged every 2–3 d, and cells were split once a week. All experiments were conducted using cells at passage 19 ± 5.

### Isolation of proteins from eukaryotic cells

Caco-2 cells were grown in cell culture medium. Medium was exchanged every 2–3 d. After 7 d, the medium was aspirated, cells were washed twice with PBS, and detached using Trypsin-EDTA (Thermo Fisher Scientific, Waltham, MA, USA). Cells were pelleted at 300 x g for 4 min, resuspended in fresh cell culture medium, and counted using the CASY Cell counter & Analyzer (OLS OMNI Life Science GmbH & Co. KG, Bremen, Germany). A total of 6 × 10^6^ cells were transferred into a reaction tube and washed twice with PBS. According to previous protocols,^32^ cells were resuspended in 100 µl RIPA buffer (Thermo Fisher Scientific, Waltham, MA, USA) supplemented with complete™ protease inhibitor cocktail (Roche, Mannheim, Germany), sonicated at 10% for 10 × 2 s at intervals of 10 s and incubated overnight at 4 °C. On the following day, samples were centrifuged at 12,000 x g and 4 °C for 10 min. The supernatant was transferred into a new reaction tube and stored at –80 °C.

### BEV isolation and purification

Bacterial cultures were centrifuged to pellet the bacterial cells. Supernatants were sterile-filtered using Millipore® Stericup® Quick Release Vacuum Filtration Systems with PVDF membranes and a pore size of 0.22 µm (*P. mirabilis*) or 0.45 µm (*L. casei* and *E. faecalis*) (Merck Millipore, Burlington, MA, USA) to remove residual bacteria. Filtered supernatants were transferred into 70 ml polycarbonate ultracentrifuge tubes (Beckman Colter, Brea, CA, USA) and centrifuged at 100,000 x g at 4 °C for 2 hours (*L. casei* and *P. mirabilis*) or at 160,000 x g at 4 °C for 3 hours (*E. faecalis*). After ultracentrifugation, the supernatants were discarded, and the pellets from three ultracentrifuge tubes were pooled and resuspended in a total volume of 500 µl filtered PBS (Thermo Fisher Scientific, Waltham, MA, USA). The resuspended pellets were either stored at –80 °C or immediately purified under aseptic conditions via size exclusion chromatography (SEC) using a column packed with 40 ml Sepharose™ CL-2B (Sigma-Aldrich, St. Louis, MO, USA) in PBS. Purification was performed in order to remove free proteins and other small contaminants that could interfere with downstream analyzes. Fractions of 1 ml each were collected, and BEVs consistently eluted in fractions 12–15 (Supplementary Figure 2A–F) as previously reported using similar experimental setups.^33-35^ Collected fractions were stored at –80 °C until further use.

### EV isolation and purification

EVs were isolated from three different conditions. Cell culture supernatant from Caco-2 cells grown in cell culture medium with supplements [Caco-2 (+FCS), conditioned medium] was collected after approximately 72 h of growth on the day of cell passaging (day 7 after seeding). Cells were pelleted at 300 x g for 4 min, and the cell-free supernatant was stored at –80 °C until further processing. For EV isolation from cells grown without supplements [Caco-2 (-FCS)], cells were initially maintained in cell culture medium with supplements for 7 d. Then, the cells were washed twice with PBS, and DMEM (Thermo Fisher Scientific, Waltham, MA, USA) without supplements was added to the cells. After 24 h at 37 °C and 5% CO_2_, the supernatant was collected, fresh DMEM (Thermo Fisher Scientific, Waltham, MA, USA) without supplements was added to the cells, and the supernatant was again collected after additional 24 h. As described above, all supernatants were centrifuged at 300 x g for 4 min to pellet the cells, and the cell-free supernatant was stored at –80 °C.

For EV isolation, cell-free supernatants were thawed at room temperature (RT) and centrifuged at 3,000 x g for 20 min at 4 °C to remove larger particles. In parallel, fresh cell culture medium with supplements [DMEM (+FCS), unconditioned medium] underwent the same centrifugation step to ensure similar initial preparation of both media. The following steps were carried out identically for all EV preparations. Supernatants were carefully transferred into 70 ml polycarbonate ultracentrifuge tubes (Beckman Colter, Brea, CA, USA) and centrifuged at 100,000 x g at 4 °C for 2 hours. After ultracentrifugation, the supernatant was discarded, and EV pellets from three ultracentrifuge tubes were pooled and resuspended in a total volume of 500 µl filtered PBS (Thermo Fisher Scientific, Waltham, MA, USA). For visualization of the interaction of bacteria with EVs, pelleted Caco-2 (-FCS) EVs were resuspended in 200 µl filtered PBS. The resuspended pellets were either stored at –80 °C or directly purified via SEC using a column packed with 40 ml Sepharose™ CL-2B (Sigma-Aldrich, St. Louis, MO, USA) in PBS. As described above, purification was performed in order to remove free proteins and other small contaminants that could interfere with downstream analyzes. Fractions of 1 ml each were collected, and EVs consistently eluted in fractions 13–15 (Supplementary Figure 2G–L). Collected fractions were stored at –80 °C until further use.

### Determination of protein content

Protein concentrations of EV and BEV fractions after purification via SEC, as well as those of the Caco-2 protein extracts, were quantified using the QuantiPro BCA Assay Kit (Sigma-Aldrich, St. Louis, MO, USA) according to the manufacturer’s recommendations.

### Endotoxin quantification

Endotoxin (lipopolysaccharide, LPS) concentrations of *P. mirabilis* OMV and *P. mirabilis* OMV-RNA samples were determined using the Pierce™ Chromogenic Endotoxin Quant Kit (Thermo Fisher Scientific, Waltham, MA, USA). Briefly, 50 µl of each OMV sample and 1 µl of each OMV-RNA sample were used, and the assay was performed according to the manufacturer’s instructions. PBS was used as a reference for determination of the endotoxin concentration in OMV samples, whereas nuclease-free water was used as a reference for determination of the endotoxin concentration in OMV-RNA samples. Results are presented as mean ± standard deviation (SD) of 3−4 independent biological replicates, each representing a separate vesicle or RNA preparation. Endotoxin concentrations were expressed as endotoxin units (EU) per milliliter and converted to nanogram per milliliter (10 EU/ml = 1 ng/ml).

### Determination of particle concentration and size

EV and BEV concentration and size distribution were measured using NanoSight (Malvern Panalytical, Malvern, Worcestershire, UK).^36^ Prior to measurement, samples were diluted with filtered PBS, and 400 µl of the diluted sample was introduced into the measuring chamber. Videos with a length of 30 s were recorded in triplicates with a camera level of 15. Particle concentration and size were calculated by NTA 3.4 software (v3.4.4), with a detection threshold set to 5. The size distribution of EVs and BEVs is presented as mean ± standard error.

### Cryo-transmission electron microscopy (Cryo-TEM)

For cryo-TEM, a 2 µl droplet of the sample was placed on a holey carbon supported copper grid (Plano, Wetzlar, Germany, type S147-4), blotted for 2 s, and vitrified in undercooled liquid ethane using a Gatan (Pleasanton, CA, USA) CP3 plunge-freezer. The frozen hydrated specimen was transferred to a Gatan model 914 cryo-TEM sample holder under liquid nitrogen and visualized using a JEOL (Akishima, Tokyo, Japan) JEM-2100 LaB_6_ TEM at 200 kV accelerating voltage under low-dose conditions (CCD camera Gatan Orius SC1000, 2 s acquisition time).

### Scanning electron microscopy (SEM)

For SEM, overnight cultures of all three bacteria were diluted to an OD of 0.1 with fresh broth in a total volume of 2 ml and grown statically at 37 °C under ambient atmospheric conditions. After 12 h (*E. faecalis* and *P. mirabilis*) or 40 h (*L. casei*) bacteria were fixed with 3% (v/v) glutardialdehyde (Thermo Fisher Scientific, Waltham, MA, USA) for 2 h at RT. Next, the samples were washed with increasing concentrations of ethanol up to 100%, and hexamethyldisilazane (HMDS) (Sigma-Aldrich, St. Louis, MO, USA) was added for the final drying process. HMDS was removed, and the samples were air-dried at RT for several hours.^37^ Dried samples were placed on sticky carbon tapes, and sputter coated with gold using Q150R ES sputter coater (Quorum Technologies Ltd., Laughton, East Sussex, UK). Images were taken with Evo HD 15 (Carl Zeiss Microscopy GmbH, Jena, Germany) and processed using SmartSEM software (v6.09) (Supplementary Figure 1D–F).

### Western blot

EV and cellular marker proteins were analyzed by Western blot. For gel electrophoresis, 15 µg Caco-2 protein extract and 30 µl of each EV sample were mixed with 2x sample buffer (130 mM Tris-HCl, 6% (v/v) SDS, 10% (v/v) 3-mercapto-1, 2-propanediol, 10% (v/v) glycerol). Samples were sonicated 3 × 3 s (40%), incubated on ice for 5 min, then heated at 99 °C for 10 min. After centrifugation at 14,000 rpm and 4 °C for 10 min, supernatants were used for further analysis. Proteins were separated using (4–15%) mini-PROTEAN® TGX™ Precast Protein Gels (Bio-Rad Laboratories Inc., Hercules, CA, USA) for 30 min at 12 mA, followed by 40 min at 25 mA. Separated proteins were transferred onto a nitrocellulose membrane (GE Healthcare, Chicago, IL, USA) for 2 h at 400 mA. Anti-Calnexin antibody (#2679, RRID:AB_2228381, Cell Signaling Technology, Danvers, MA, USA) and anti-CD81 antibody (#56039, RRID:AB_2924772, Cell Signaling Technology, Danvers, MA, USA) were diluted in 5% (w/v) BSA in 1x TBST (1:800) and used to detect the protein level in Caco-2 protein extracts and EV samples. A secondary anti-rabbit antibody was purchased from Sigma-Aldrich (A0545, RRID:AB_257896). Signals were captured using the ChemiDoc™ Touch Imaging System (Bio-Rad Laboratories Inc., Hercules, CA, USA).

### BEV- and EV-RNA isolation and quality control

Total RNA was isolated from 400 µl BEV or EV fraction with the highest particle concentration using the miRNeasy Serum/Plasma Kit (Qiagen, Hilden, Germany) according to the manufacturer’s instructions, with minor modifications. During isolation of EV-RNA, 2 µl of 20 µg/µl UltraPure™ Glycogen (Thermo Fisher Scientific, Waltham, MA, USA) was added to the aqueous phase to improve RNA precipitation and increase RNA yield.^38^ Similarly, during BEV-RNA isolation, 2 µl of 20 µg/µl UltraPure™ Glycogen (Thermo Fisher Scientific, Waltham, MA, USA) was added to the aqueous phase, and an additional on-column DNase digestion using the RNA-free DNase Set (Qiagen, Hilden, Germany) was performed to eliminate potential contaminating DNA. BEV-RNA concentration was measured using the Qubit™ RNA HS Assay Kit (Thermo Fisher Scientific, Waltham, MA, USA) to enable accurate quantification of low RNA amounts, and EV-RNA concentration was measured using a NanoDrop™ 2000 spectrophotometer (Thermo Fisher Scientific, Waltham, MA, USA) as a standard method. BEV- and EV-RNA fragments were analyzed using the Agilent RNA 6000 Pico Kit for Agilent 2100 Bioanalyzer (Agilent Technologies, Santa Clara, CA, USA).

### Eukaryotic cell viability and cytotoxicity assay

Cell viability and cytotoxicity were tested after incubation with BEVs or after transfection with BEV-RNA. Caco-2 cells were seeded with a density of 7.5 × 10^4^ cells/well in a 96-well plate and incubated at 37 °C and 5% CO_2_. After 24 h, the medium was aspirated and replaced with 100 µl fresh FCS-free medium, together with 100 µl of either the BEV sample, one of three serial 1:10 dilutions of the BEVs to establish a dose–response relationship, or 0.2 ng/ml *P. mirabilis* LPS (Sigma-Aldrich, St. Louis, MO, USA). Detailed information on particle/cells ratios can be found in Supplementary Table 1. Every experiment included a live-control using cells incubated with filtered PBS, and a dead-control using cells treated with 1% (v/v) Triton X-100 (Sigma-Aldrich, St. Louis, MO, USA).^35^ To test the cell viability after transfection with BEV-RNA, different amounts of BEV-RNA or 5 µl of 1 ng/ml *P. mirabilis* LPS (Sigma-Aldrich, St. Louis, MO, USA) were mixed with the transfection reagent Lipofectamine™ 3000 (Thermo Fisher Scientific, Waltham, MA, USA) according to the manufacturer’s instructions and added to the cells along with fresh FCS-free medium to a final volume of 200 µl. Detailed information on BEV-RNA amounts can be found in Supplementary Table 1. Controls included cells incubated with transfection reagent mixed with nuclease-free water (vehicle), and cells treated with 1% (v/v) Triton X-100 (Sigma-Aldrich, St. Louis, MO, USA).

After an additional 24 h or 48 h, cytotoxicity was assessed using the Cytotoxicity Detection Kit (Roche, Mannheim, Germany). Briefly, 100 µl of supernatant was mixed with 100 µl LDH working solution. After incubation at RT for 5 min under mild shaking, the absorbance was measured at a wavelength of *λ* = 490 nm. To further test the viability of the cells, PrestoBlue reagent (Thermo Fisher Scientific, Waltham, MA, USA) was diluted 1:10 with fresh medium. The remaining medium in the 96-well plate was aspirated, and 100 µl of diluted PrestoBlue reagent was added carefully to the cells. After incubation for 20 min at 37 °C, the fluorescence (λEx/λEm 560/590 nm) was measured. Results are presented as mean ± SD of 3−4 independent biological replicates. Statistical significance was calculated in GraphPad Prism 8 (v8.4.3) using a two-tailed unpaired t-test, with *p* < 0.05 considered statistically significant.

### Flow cytometry and confocal laser scanning microscopy (CLSM) of eukaryotic cells

BEVs were labeled according to previous protocols.^39^ In brief, unpurified BEV pellets were incubated with 2 µl (*L. casei*) or 1 µl (*E. faecalis* and *P. mirabilis*) Vybrant™ DiI (Thermo Fisher Scientific, Waltham, MA, USA) for 30 min at 37 °C. Labeled BEVs were purified via SEC using a column packed with 40 ml Sepharose™ CL-2B (Sigma-Aldrich, St. Louis, MO, USA) in PBS to remove free proteins and unbound dye. Based on prior analysis, fractions containing BEVs were collected. 50 µl of each collected fraction was used to measure the fluorescence (λEx/λEm 490/570 nm). The fraction with the highest fluorescence signal was selected for further use.

For flow cytometry, cells were seeded in 48-well plates at 2 × 10^5^ cells/well and incubated at 37 °C and 5% CO_2_. After 24 h, the cell culture medium was aspirated. Stained BEVs or PBS as a control were diluted 1:5 with fresh cell culture medium and added to the cells. After 4 h, 12 h, 24 h, or 48 h, the supernatant was removed, the cells were washed with PBS, and detached using Trypsin-EDTA (Thermo Fisher Scientific, Waltham, MA, USA). Cells were fixed with 4% (v/v) formaldehyde (Thermo Fisher Scientific, Waltham, MA, USA) for 30 min at RT, washed once with PBS, and resuspended in 600 µl PBS. To detect DiI, a laser at 561 nm (PE, Phycoerythrin) was used (BD LSRFortessa™ Cell Analyzer, Becton Dickinson, Franklin Lakes, NJ, USA). A total of 10,000 events per sample was set to be analyzed by BD FACSDiva™ software (v9.0.1) and evaluated by FlowJo™ software (v10.6.0). Results are presented as mean ± SD of 3−4 independent biological replicates.

For CLSM, the cells were seeded in 8-well chamber slides (Thermo Fisher Scientific, Waltham, MA, USA) with a density of 2 × 10^5^ cells/well and incubated at 37 °C and 5% CO_2_. After 24 h, the cell culture medium was aspirated, and stained BEVs or PBS as a control were diluted 1:5 with fresh cell culture medium and added to the cells. After 12 h, 24 h, or 48 h, cells were washed with PBS and fixed with 4% (v/v) formaldehyde (Thermo Fisher Scientific, Waltham, MA, USA) for 20 min at RT. After fixation, cells were either analyzed directly or permeabilized, and unspecific binding sites were blocked using 1% (w/v) bovine serum albumin/0.05% (w/v) saponin for 20 min at RT. F-actin was stained using Alexa Fluor™ 488 Phalloidin (Thermo Fisher Scientific, Waltham, MA, USA) for 20 min, and cell nuclei were stained using 0.5 µg/ml 4′, 6-diamidino-2-phenylindole dihydrochloride (DAPI) (Sigma-Aldrich, St. Louis, MO, USA) for another 20 min at RT.^40^ Wash steps with PBS were performed between the different staining steps. For confocal imaging (Leica TCS SP8 System, Leica Microsystems, Wetzlar, Germany) a laser at 405 nm was used to visualize DAPI, a laser at 488 nm for Alexa Fluor™ 488 Phalloidin, and a laser at 561 nm for DiI. The images were captured and processed using Leica Application Suite X software.

### Transcriptome analysis of eukaryotic cells

Caco-2 cells were seeded in 48-well plates with a density of 1.2 × 10^5^ cells/well and incubated at 37 °C and 5% CO_2_. After 24 h, medium was aspirated, and BEVs or BEV-RNA mixed with transfection reagent Lipofectamine™ 3000 (Thermo Fisher Scientific, Waltham, MA, USA) were added along with fresh medium (Supplementary Table 1). Controls for cells incubated with BEVs included PBS and 0.2 ng/ml *P. mirabilis* LPS (Sigma-Aldrich, St. Louis, MO, USA). Controls for cells transfected with BEV-RNA included 100 ng, 5 ng, or 2 ng of AllStars Negative Control siRNA (ANC) (Qiagen, Hilden, Germany), or 5 µl of 1 ng/ml *P. mirabilis* LPS (Sigma-Aldrich, St. Louis, MO, USA) mixed with Lipofectamine™ 3000 transfection reagent. After 10 h, 24 h, or 48 h, supernatants were aspirated, and the cells were washed once with PBS. Subsequently, the cells were lysed by adding 700 µl Qiazol Lysis Reagent (Qiagen, Hilden, Germany) to each well and stored at –80 °C until RNA isolation. Total RNA was isolated from the Caco-2 cells using the miRNeasy Micro Kit (Qiagen, Hilden, Germany) according to the manufacturer’s instructions. RNA concentration was measured using a NanoDrop™ 2000 spectrophotometer (Thermo Fisher Scientific, Waltham, MA, USA) and RNA integrity of selected samples was determined using Agilent RNA 6000 Nano Kit for Agilent 2100 Bioanalyzer (Agilent Technologies, Santa Clara, CA, USA).

DNA libraries were prepared on the high-throughput MGISP-960 sample preparation system using the MGIEasy rRNA Depletion Kit and MGIEasy RNA Library Prep Set (MGI Tech Co. Ltd., Shenzhen, Guangdong, China) according to the manufacturer’s recommendations. Concentrations of the PCR products were determined using Qubit™ 1x dsDNA HS Assay Kit (Thermo Fisher Scientific, Waltham, MA, USA). After PCR, different barcoded samples were pooled in equal amounts and circularized to generate single-stranded DNA libraries (ssDNA). The concentration of the ssDNA libraries was measured using Qubit™ ssDNA Assay Kit (Thermo Fisher Scientific, Waltham, MA, USA). DNA nanoballs (DNB) were generated by rolling circle amplification and paired-end sequencing (PE100) was performed using DNBSEQ-G400RS High-throughput Sequencing Reagent Set on a DNBSEQ-G400 instrument (MGI Tech Co. Ltd., Shenzhen, Guangdong, China).

### EV miRNome analysis

DNA libraries were prepared using the MGIEasy smallRNA Library Prep Kit (MGI Tech Co. Ltd., Shenzhen, Guangdong, China) according to the manufacturer’s recommendations. In brief, 100 ng of RNA from conditioned [Caco-2 (+FCS), *n* = 9] or unconditioned [DMEM (+FCS), *n* = 8] medium EVs were used as input. Amplified PCR products were separated using 6% Novex™ TBE gels (Thermo Fisher Scientific, Waltham, MA, USA). The target band from 100 to 120 bp was cut out and PCR products were eluted from the gel and purified by ethanol precipitation.^41^ Concentrations of the PCR products were determined using Qubit™ 1x dsDNA HS Assay Kit (Thermo Fisher Scientific, Waltham, MA, USA). Next, different barcoded samples were pooled in equal amounts and circularized to generate single-stranded DNA libraries (ssDNA). Concentration of the ssDNA libraries was measured using Qubit™ ssDNA Assay Kit (Thermo Fisher Scientific, Waltham, MA, USA). DNA nanoballs (DNB) preparation and single-end sequencing (SE50) was carried out as a service at BGI in Shenzhen, China.

### Measurement of bacterial growth and viability

To assess bacterial growth after incubation with EVs, overnight cultures for all three bacteria were prepared. On the following day, the OD was measured, and cultures were diluted to 0.01 with fresh broth. EVs, two serial 1:10 dilutions of the EVs, which were used to establish a dose–response relationship, or PBS as a control, were diluted 1:1 with bacterial suspension to a total volume of 200 µl. Cultures were grown statically at 37 °C under ambient atmospheric conditions, and growth was monitored by measuring the OD for 36 h or 48 h.

For analysis of bacterial growth and viability after incubation with synthetic miR-192-5p (Eurofins Genomics Germany GmbH, Ebersberg, Germany), overnight cultures for all three bacteria were prepared. On the following day, the OD was measured, and cultures were diluted to 0.01 with fresh broth. Synthetic miR-192-5p was added at 0.5 µM, 2 µM, or 4 µM, or PBS as a control to a total volume of 150 µl. Cultures were grown statically at 37 °C under ambient atmospheric conditions, and growth was monitored by measuring the OD for 36 h or 48 h. Results are presented as mean ± SD of 4 independent biological replicates. Bacterial viability was analyzed after 24 h (*E. faecalis* and *P. mirabilis*) or 40 h (*L. casei*) of growth in the presence of 4 µM synthetic miR-192-5p or 1x siRNA Max Buffer (supplied by Eurofins Genomics Germany GmbH to resuspend the synthetic miR-192-5p) using Live/Dead™ BacLight™ Bacterial Viability Kit (Thermo Fisher Scientific, Waltham, MA, USA) according to the manufacturer’s recommendations. In brief, bacteria were stained with SYTO™ 9/PI for 15 min at RT, and the fluorescence of SYTO™ 9 (λEx/λEm 485/530 nm) and PI (λEx/λEm 485/630 nm) was measured. The percentage of live bacteria was determined using the adjusted dye ratio equation by Ou et al.^42^.

For analysis of bacterial viability after incubation with liposome (Lipofectamine™ 3000-based lipid complexes)-packaged synthetic miR-192-5p, overnight cultures for all three bacteria were prepared. The OD was measured, and cultures were diluted to 0.01 with fresh broth. Next, 4 µM of synthetic miR-192-5p was mixed with Lipofectamine™ 3000 (Thermo Fisher Scientific, Waltham, MA, USA) and added to a total volume of 150 µl. Controls included bacteria incubated with DMEM, or Lipofectamine mixed with either 1x siRNA Max Buffer (supplied by Eurofins Genomics Germany GmbH to resuspend the synthetic miR-192-5p) or 8 µg polyadenylic acid (Sigma-Aldrich, St. Louis, MO, USA). Bacterial viability was analyzed after 24 h (*E. faecalis* and *P. mirabilis*) or 40 h (*L. casei*) of growth using Live/Dead™ BacLight™ Bacterial Viability Kit (Thermo Fisher Scientific, Waltham, MA, USA) as described above. Results of bacterial viability analyzes are presented as mean ± SD of 4 independent biological replicates. For all comparisons, statistical significance was calculated in GraphPad Prism 8 (v8.4.3) using a two-tailed unpaired t-test, with *p* < 0.05 considered statistically significant.

### Flow cytometry and confocal laser scanning microscopy (CLSM) of bacteria

For analysis of the interaction of bacteria with EVs, vesicles were labeled with PKH26 (Sigma-Aldrich, St. Louis, MO, USA). PKH26 was diluted 1:250 (v/v) in Diluent C. Next, 200 µl unpurified Caco-2 (-FCS) EV pellet was incubated with 200 µl diluted PKH26 for 10 min at RT.^43^ Labeled EVs were purified via SEC using a column packed with 40 ml Sepharose™ CL-2B (Sigma-Aldrich, St. Louis, MO, USA) in PBS to remove free proteins and unbound dye. Based on prior analysis, fractions containing EVs were collected. 50 µl of each collected fraction was used to measure the fluorescence (λEx/λEm 490/570 nm). The fraction with the highest fluorescence signal was used for further experiments. Overnight cultures of all three bacteria were diluted to OD 0.01 with fresh broth and labeled EVs or PBS as a control were mixed 1:1 with bacterial suspension to a total volume of 200 µl and grown statically at 37 °C under ambient atmospheric conditions. As previously described,^44^ after 4 h, 8 h or 24 h, bacteria were stained with SYTO™ 9 for 10 min at 37 °C, fixed with 4% (v/v) formaldehyde (Thermo Fisher Scientific, Waltham, MA, USA) for 10 min at 37 °C, washed once, and resuspended in PBS. 5 µl of each sample was applied on a coverslip together with a drop of fluorescence mounting medium (Agilent Technologies, Santa Clara, CA, USA). For confocal imaging (Leica TCS SP8 System, Leica Microsystems, Wetzlar, Germany) a laser at 488 nm was used to visualize SYTO™ 9, and a laser at 561 nm for PKH26. The images were captured and processed using Leica Application Suite X software.

For analysis of the interaction of bacteria with miRNAs, a Cy3-labeled synthetic miR-192-5p (Eurofins Genomics, Germany GmbH, Ebersberg, Germany) was used. Overnight cultures of all three bacteria were diluted to OD 0.01 with fresh broth, and 4 µM of synthetic miR-192-5p in PBS, DMEM, or mixed with Lipofectamine™ 3000 (Thermo Fisher Scientific, Waltham, MA, USA) was added to a total volume of 150 µl. Controls included bacteria incubated with PBS, DMEM, or Lipofectamine™ 3000 mixed with 1x siRNA Max Buffer (supplied by Eurofins Genomics Germany GmbH to resuspend the synthetic miR-192-5p). As described above, after 4 h, 8 h, 24 h, or 48 h, bacteria were stained with SYTO™ 9 for 10 min at 37 °C, fixed with 4% (v/v) formaldehyde (Thermo Fisher Scientific, Waltham, MA, USA) for 10 min at 37 °C, washed once, and resuspended in PBS. 5 µl of each sample was applied on a coverslip together with a drop of fluorescence mounting medium (Agilent Technologies, Santa Clara, CA, USA). For confocal imaging (Leica TCS SP8 System, Leica Microsystems, Wetzlar, Germany) a laser at 488 nm was used to visualize SYTO™ 9, and a laser at 561 nm for Cy3. The images were captured and processed using Leica Application Suite X software. The remaining sample volume was used for flow cytometry. To detect Cy3, a laser at 561 nm (PE, Phycoerythrin), and to detect SYTO™ 9, a laser at 488 nm (Alexa Fluor 488) was used (BD LSRFortessa™ Cell Analyzer, Becton Dickinson, Franklin Lakes, NJ, USA). A total of 100,000 events (*E. faecalis*) or 200,000 events (*L. casei* or *P. mirabilis*) per sample was set to be analyzed by BD FACSDiva™ software (v9.0.1) and evaluated by FlowJo™ software (v10.6.0). Results are presented as mean ± SD of 4 independent biological replicates. Statistical significance was calculated in GraphPad Prism 8 (v8.4.3) using a two-tailed unpaired t-test, with *p* < 0.05 considered statistically significant.

### Data analysis

Snakepipes pipeline (v2.7.3)^45^ was used to process the paired-end Fastq files. Multiqc (v1.12)^46^ was used to carry out initial QC. STAR (v2.7.10b)^47^ was used to align the reads against GRCh38 using default options. Deduplication was performed with the markdup function from sambamba (v.0.8.0).^48^ FeatureCount from subread (v.2.0.1)^49^ was used to quantify mRNA counts with the parameters -C -Q 10 --primary. The raw counts were used to perform differential expression analysis using DESeq2 (v1.30.1).^50^ Alongside the fold-change values provided by DESeq2, we calculate Cohen's *d* using the variance stabilizing transform normalized counts created by Deseq2. We selected deregulated genes based on an absolute Log2 FC > 1 and an absolute Cohen's *d* > 0.8. The resulting lists were used to calculate gene set overlaps. GeneTrail 3.2^51^ was used for pathway analysis of differentially expressed genes by performing an over-representation analysis (ORA) with the following setting [Null hypothesis (for *p*-value computation): Two-sided; Method to adjust *p*-values: Benjamini-Hochberg; Significance level: 1; Minimal size of category: 1; reference set: all supported genes].

For miRNAs, miRmaster 2.0 pipeline^52^ was used to trim, collapse, and quantify the single-end sequencing Fastq files using miRBase 22.1.^53^ Raw counts were used for differential expression analysis using DESeq2 and *p*-values were adjusted by a Benjamini-Hochberg correction with a false discovery rate (FDR) of 10%. For creating the heatmap, the top 25 most highly variable miRNAs were taken and clustered using ComplexHeatmap package (v2.22.0).^54^ The counts were normalized using reads per million mapped miRNA (rpmmm) for detailed visualization.

Snakemake (v8.2.3),^55^ Python (v3.12.2), matplotlib (v3.9.1),^56^ seaborn (v0.13.2),^57^ upsetplot (v0.9.0), R (v4.0.2), ggplot2 (v3.3.6)^58^ and GraphPad Prism 8 (v8.4.3) were used for calculations and plotting.

## Results

### *Lacticaseibacillus casei*, *Enterococcus faecalis* and *Proteus mirabilis* produce BEVs and Caco-2 cells can interact with the BEVs

**Figure 1.**
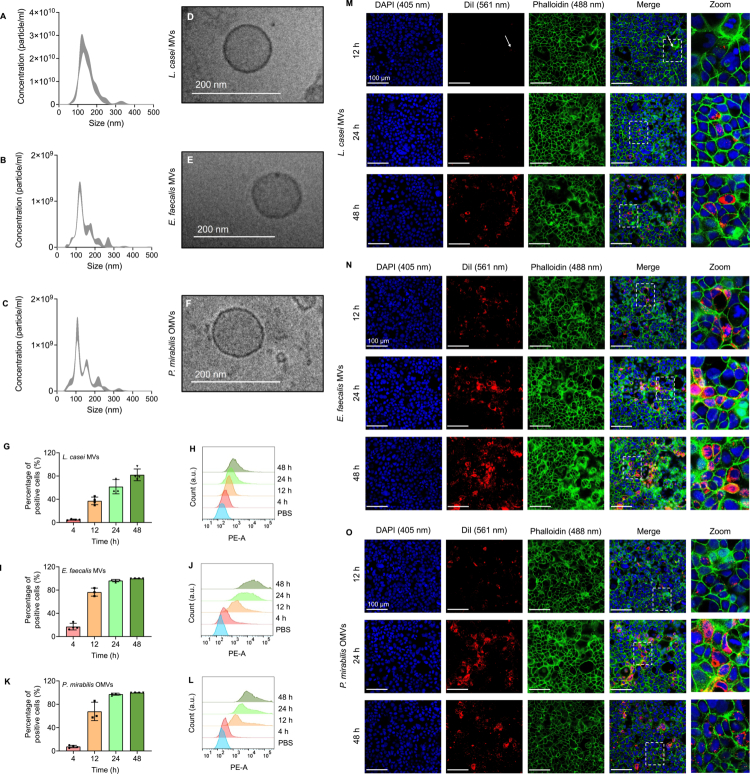
Interaction of Caco-2 cells with BEVs from *L. casei*, *E. faecalis* and *P. mirabilis*. (A–F) Characterization of BEVs. Typical size distribution of purified BEVs from *L. casei* MVs (A), *E. faecalis* MVs (B), and *P. mirabilis* OMVs (C) measured by nanoparticle tracking analysis (NTA). Data are shown as mean ± standard error. Representative cryo-TEM images of *L. casei* MVs (D), *E. faecalis* MVs (E), and *P. mirabilis* OMVs (F) showed round shaped particles. (G–L) Flow cytometry analysis of Caco-2 cells after incubation with DiI-labeled BEVs. Shown is the percentage of DiI-positive cells and representative histograms compared to the control (PBS) after 4 h, 12 h, 24 h, or 48 h incubation with the DiI-labeled BEVs from *L. casei* (G and H), *E. faecalis* (I and J), or *P. mirabilis* (K and L). Results are presented as mean ± standard deviation (SD) from 3−4 independent biological replicates. (M–O) Visualization of the interaction of Caco-2 cells with BEVs cells using CLSM. Caco-2 cells were incubated with DiI-labeled *L. casei* MVs (M), *E. faecalis* MVs (N), or *P. mirabilis* OMVs (O) for 12 h, 24 h, or 48 h. After the incubation, cells were washed, fixed, and permeabilized. F-actin was stained with Alexa Fluor™ 488 Phalloidin, and cell nuclei were stained with DAPI. DAPI was visualized using a laser at 405 nm (blue), Alexa Fluor™ 488 Phalloidin with a laser at 488 nm (green), and DiI with a laser at 561 nm (red). The white arrow indicates an area where weak fluorescence signals for DiI-labeled *L. casei* MVs were detected. Scale bar: 100 µm.

To study the effect of BEVs on eukaryotic recipient cells, we first isolated BEVs from bacterial cultures using ultracentrifugation followed by purification of BEVs via SEC. The isolated BEVs were then characterized using nanoparticle tracking analysis (NTA) and cryo-TEM. Analyzing BEVs from each bacterium individually, we found that *L. casei* MVs had a mode size of 140.0 (±15.5) nm (Figure 1A), *E. faecalis* MVs of 117.1 (±7.5) nm (Figure 1B), and *P. mirabilis* OMVs of 115.2 (±7.4) nm (Figure 1C). Additionally, the particle concentration was found to be 2.1 × 10^12^ (±6.4 × 10^11^) particles/ml for *L. casei* MVs, 8.5 × 10^10^ (±8.2 × 10^9^) particles/ml for *E. faecalis* MVs, and 6.4 × 10^10^ (±1.0 × 10^10^) particles/ml for *P. mirabilis* OMVs. Cryo-TEM images of BEVs showed round-shaped particles (Figure 1D–F). Since OMVs from Gram-negative bacteria typically contain LPS,^59^ we additionally quantified the LPS concentration of *P. mirabilis* OMVs, demonstrating that OMVs contained approximately 0.2 ng/ml LPS (Supplementary Figure 3A).

Next, we asked whether Caco-2 cells can interact with BEVs from *L. casei*, *E. faecalis,* and *P. mirabilis.* Using flow cytometry, we found that among the three BEV types, Caco-2 cells incubated with DiI-labeled *L. casei* MVs showed the lowest percentage of fluorescence-positive cells, with a maximum of 82.1 (±10.1) % fluorescent cells after 48 h (Figure 1G and H). In contrast, after 24 h incubation with labeled *E. faecalis* MVs or *P. mirabilis* OMVs, we detected 96.3 (±2.0) % and 97.3 (±1.8) % fluorescent cells (Figure 1I–L). To further assess the localization of DiI-labeled BEVs, we used confocal laser scanning microscopy (CLSM). In accordance with the previous results, we observed only little fluorescent signals for *L. casei* MVs within the cells after 12 h, which increased with extended incubation. In comparison, for cells incubated with *E. faecalis* MVs or *P. mirabilis* OMVs we observed higher fluorescence signals from the particles already after 12 h incubation. The signals intensified over time and appeared to accumulate within the Caco-2 cells (Figure 1M–O). We further observed that washing with PBS during the staining procedure led to a minor removal of fluorescent particles mostly after 12 h of incubation. Accordingly, a slightly higher number of fluorescently labeled particles was detected when cells were analyzed directly after fixation without additional staining and washing steps (Supplementary Figure 4). This suggests that some BEVs may have been bound to the cell surface. However, we did not observe this removal with longer incubation time, which is consistent with the idea of an apparent uptake of the BEVs into the cells.

### BEVs contain distinct RNA amounts and species, and incubation or transfection of Caco-2 cells with BEVs or BEV-RNA display no cytotoxic effects

**Figure 2.**
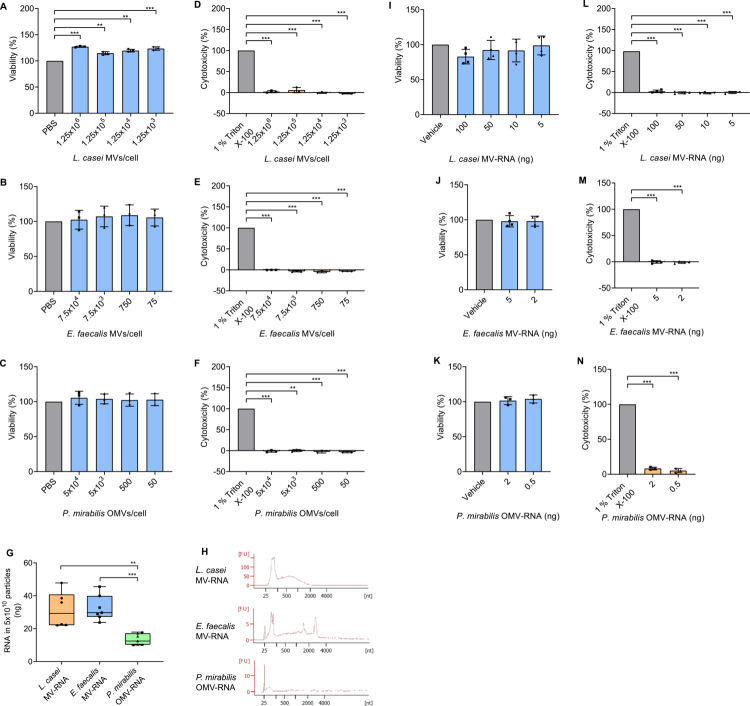
Effects of BEVs and BEV-RNA on Caco-2 cell viability. (A–F) Measurement of cell viability and cytotoxicity after incubation of Caco-2 cells with different concentrations of *L. casei* MVs (A and D), *E. faecalis* MVs (B and E), or *P. mirabilis* OMVs (C and F) for 24 h (*E. faecalis* MVs or *P. mirabilis* OMVs) or 48 h (*L. casei* MVs). 1% Triton X-100 was used as dead control, whereas PBS was used as live control. Results are presented as mean ± SD from 3 independent biological replicates. (G and H) Analysis of BEV-derived RNA. (G) Box-whisker-plots show the RNA amounts isolated from equal BEV sample volumes and subsequently recalculated and normalized to 5 × 10^10^ particles based on NTA-derived particle concentrations. Data represent 6−7 independent RNA preparations. (H) Representative electropherograms shows the size distribution of *L. casei* MV-RNA, *E. faecalis* MV-RNA, and *P. mirabilis* OMV-RNA, analyzed using Agilent RNA 6000 Pico Chip. (I–N) Measurement of cell viability and cytotoxicity after transfection of Caco-2 cells with different amounts of *L. casei* MV-RNA (I and L), *E. faecalis* MV-RNA (J and M), or *P. mirabilis* OMV-RNA (K and N) for 24 h (*E. faecalis* MV-RNA or *P. mirabilis* OMV-RNA) or 48 h (*L. casei* MV-RNA). 1% Triton X-100 was used as dead control, whereas Lipofectamine™ 3000 transfection reagent mixed with nuclease-free water (vehicle) was used as live control. Results are presented as mean ± SD from 3-4 independent biological replicates. Statistical significance was determined using a two-tailed unpaired t-test (*p* < 0.01**, *p* < 0.001***).

To investigate whether exposure to BEVs affects the viability of Caco-2 cells, we incubated the cells with different concentrations of BEVs for 24 h or 48 h and performed viability and cytotoxicity assays. None of the BEVs showed a negative impact on cell viability or increased cytotoxicity at any of the concentrations tested (Figure 2A–F). Notably, incubation of Caco-2 cells with *L. casei* MVs resulted in a significant increase (two-tailed unpaired t-test, *p* < 0.01) in cell viability compared to the control (Figure 2A). This effect was not observed with BEVs from the other two bacterial species (Figure 2B and C). Additionally, incubation of Caco-2 cells with LPS from *P. mirabilis* alone did not affect the cell viability (Supplementary Figure 3B and 3C).

Given that BEV-associated RNA was found to modulate the cellular response of host cells,^11^^,^^15^^,^^16^ we asked whether this also applies to RNA derived from *L. casei* MVs, *E. faecalis* MVs or *P. mirabilis* OMVs. We thus isolated and quantified the RNA content of BEVs. RNA was isolated from equal volumes of the BEV samples. Based on NTA-derived particle concentrations, the RNA amount was subsequently recalculated and normalized to 5 × 10^10^ particles. The resulting RNA yield was comparable for *L. casei* MVs with 31.5 (±10.9) ng and *E. faecalis* MVs with 32.6 (±7.6) ng. In contrast, *P. mirabilis* OMVs contained significantly less RNA (two-tailed unpaired t-test, *p* < 0.01) with 13.4 (±3.4) ng RNA in the same number of particles (Figure 2G). Further characterization of RNA species revealed differential cargo composition, with a high proportion of small RNAs (<200 nts) alongside larger RNAs up to approximately 2,000 nts in *L. casei* MVs. *E. faecalis* MVs contained small RNAs, larger RNA fragments, and uniquely ribosomal RNAs. In contrast, *P. mirabilis* OMVs exclusively carried small RNAs (Figure 2H). In summary, our results demonstrate that BEVs isolated from different bacterial species exhibit diverse RNA cargo profiles. We further quantified the LPS concentration in the RNA isolated from *P. mirabilis* OMVs and found that the RNA contained approximately 1 ng/ml LPS (Supplementary Figure 3D).

To assess potential cytotoxic effects of BEV-RNA, Caco-2 cells were transfected with different amounts of BEV-RNA for 24 h or 48 h, followed by viability and cytotoxicity assays. The amounts tested were chosen based on the amount of RNA associated with the BEVs that could potentially be delivered to recipient cells. Consistent with the incubation of BEVs, we did not observe compromised cell viability or increased cytotoxicity at any of the tested amounts of BEV-RNA (Figure 2I–N). Similarly, transfection with *P. mirabilis* LPS alone had no impact on cell viability (Supplementary Figure 3E and 3F).

### The transcriptome of Caco-2 cells changes in response to incubation with BEVs or transfection with BEV-RNA

**Figure 3.**
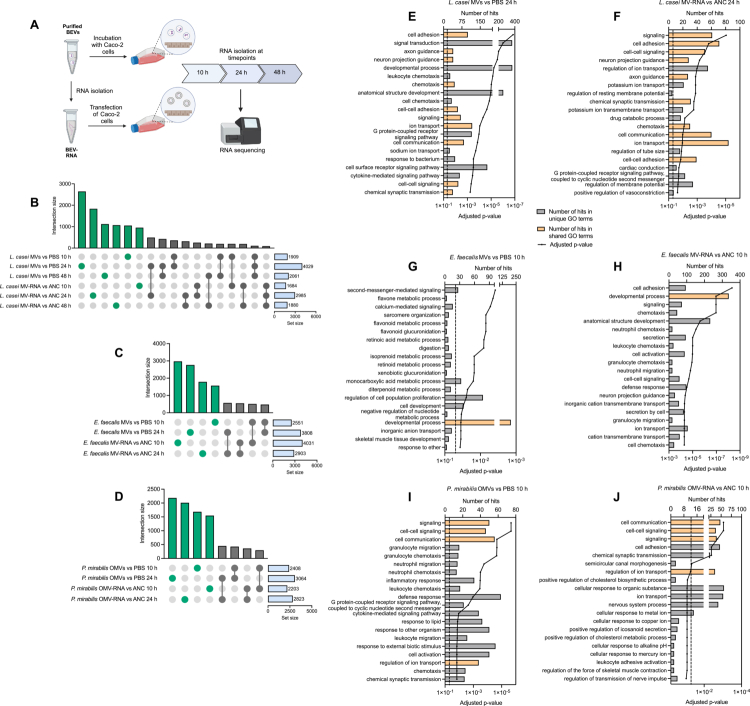
Changes in the gene expression of Caco-2 cells after incubation with BEVs or transfection with BEV-RNA. (A) Illustration of the experimental workflow during transcriptome analysis (Created with BioRender). Caco-2 cells were incubated with BEVs (*L. casei* MVs: 9.4 × 10^5^ particles/cell, *E. faecalis* MVs: 4.7 × 10^4^ particles/cell, *P. mirabilis* OMVs: 3.75 × 10^4^ particles/cell) or transfected with BEV-RNA (*L. casei* MV-RNA: 100 ng, *E. faecalis* MV-RNA: 5 ng, *P. mirabilis* OMV-RNA: 2 ng) for 10 h, 24 h, or 48 h. (B–D) Upset plots show the intersection of differentially expressed genes between the different time points and treatments. (E–J) Differentially expressed genes were used to perform an over-representation analysis (ORA) using GeneTrail 3.2. Displayed are the top 20 Gene Ontology (GO) - biological process in which the differentially expressed genes were enriched in. The dashed line marks the significance level of Benjamini-Hochberg-adjusted *p* = 0.05. Biological processes that showed an enrichment after incubation with BEVs and transfection of BEV-RNA derived from the same bacteria at the same time point are highlighted in orange.

To examine transcriptomic changes in Caco-2 cells upon exposure with BEVs or BEV-derived RNA, we performed RNA-seq. We incubated Caco-2 cells with the different BEVs or transfected them with BEV-RNA for 10 h, 24 h, and 48 h, with 48 h time point included exclusively for *L. casei* MVs and MV-RNA due to slower interaction of the Caco-2 cells with the MVs, as determined by flow cytometry. Based on our previous results, we optimized the particles/cell ratio (Supplementary Table 1) to ensure sufficient interaction of the cells with BEVs without compromising cell viability. Notably, BEV-RNA used for transfection of Caco-2 cells was always isolated from the same BEV preparations employed in the respective incubation experiment (Figure 3A).

Across a total of 105 samples, we analyzed 3−4 samples for each time point and condition. We sequenced a median of 124 million reads (IQR: 54 million reads), of which a median of 98.2% were mapped. After filtering, we obtained a median of 107 million reads (IQR: 48 million reads). From these we assigned a median of 4.9 million reads (IQR: 2.1 million reads). After quantification, we detected a median of 36,175 genes (IQR: 1086) across the conditions, with a total of 46,395 genes detected across all conditions (Supplementary Table 2).

Following incubation of Caco-2 cells with BEVs, we observed that most of the genes, including protein-coding genes, pseudogenes, and non-coding RNAs like miRNAs, were altered after 24 h incubation (Supplementary Figure 5A–C). Interestingly, after 48 h of incubation with *L. casei* MVs, the total number of differentially expressed genes decreased, even though flow cytometry showed a continuous association of the cells with the MVs. Upon transfection of the Caco-2 cells with *L. casei* MV-RNA and *P. mirabilis* OMV-RNA, we again observed that most genes were altered after 24 h. In contrast, transfection with *E. faecalis* MV-RNA resulted in greater gene deregulation after 10 h compared to 24 h (Supplementary Figure 5D–F). Although we saw a consistent pattern in the overall deregulation after the different treatments and time points, this alone does not clarify whether the same genes were affected. Therefore, we examined the overlaps of differentially expressed genes. While many genes were uniquely deregulated depending on time point or condition, a smaller subset of genes showed shared deregulation across multiple conditions or time points. This suggests that even though the BEVs and BEV-RNA seem to cause a rather short acute deregulation of specific genes, similar events may happen which cause a change in the expression of similar genes (Figure 3B–D).

To identify biological pathways associated with the deregulated genes, we performed an over-representation analysis (ORA) using GeneTrail 3.2.^51^ For cells incubated with *L. casei* MVs for 10 h or 24 h, we found that the deregulated genes were enriched in pathways related to inflammatory response, chemotaxis, and cell adhesion (Figure 3E, Supplementary Figure 6A, Supplementary Table 3). Notably, after 48 h, no significant enrichment of biological processes related to chemotaxis were observed, which could be an indication for an attenuated cellular response to *L. casei* MVs over time. Similarly, we observed that genes deregulated after 24 h of transfection with *L. casei* MV-RNA were enriched in pathways such as chemotaxis and cell adhesion (Figure 3F, Supplementary Figure 6B–D, Supplementary Table 3), indicating that the MV-RNA alone can trigger transcriptional changes in similar pathways as the MVs themselfs. When analyzing the expression of specific genes upon incubation with *L. casei* MVs or transfection with the MV-RNA, we furthermore observed deregulation of genes such as *CCL20*, *CXCL8*, *CXCL10*, *CXCL11, IL1B, and IL6,* as well as *NOD2*, *NLRP1*, *NLRP9*, *TLR3,* and *TLR7* (Supplementary Table 4). In Caco-2 cells incubated with *E. faecalis* MVs, we observed no enrichment in biological processes related to an altered immune response after 10 h and only minor changes in genes related to inflammatory response or cell adhesion after 24 h (Figure 3G, Supplementary Figure 6E, Supplementary Table 3). In contrast, transfection of *E. faecalis* MV-RNA led to distinct transcriptional changes after already 10 h, affecting processes like defense response, cell adhesion, and chemotaxis (Figure 3H, Supplementary Figure 6F, Supplementary Table 3). Thus, it seems that the *E. faecalis* MV-RNA has a strong influence on gene expression of Caco-2 cells. For cells incubated with *P. mirabilis* OMVs for 10 h and 24 h, the differentially expressed genes were again enriched in biological processes including inflammatory response, cell adhesion, and chemotaxis (Figure 3I, Supplementary Figure 6G, Supplementary Table 3). Transfection of *P. mirabilis* OMV-RNA affected a few similar pathways, suggesting that the OMV-RNA alone can induce transcriptional changes. However, the enrichment in biological processes related to inflammatory response was less pronounced (Figure 3J, Supplementary Figure 6H, Supplementary Table 3). Again, when analyzing the expression of specific genes after incubation of the Caco-2 cells with BEVs from *E. faecalis* or *P. mirabilis*, or transfection with their respective BEV-RNA, we observed a deregulation of genes such as *CCL20*, *CXCL8,* or *CXCL11*, as well as *NOD2*, *NLRP1*, *TLR3,* or *TLR7* (Supplementary Table 4). In summary, our results show that both BEVs and BEV-derived RNA are able to cause transcriptomic changes in Caco-2 cells.

Given the presence of LPS in both *P. mirabilis* OMVs and OMV-RNA, we additionally asked to which extent LPS contributes to the observed transcriptomic changes in Caco-2 cells. Our results revealed that incubation or transfection of Caco-2 cells with *P. mirabilis* LPS led to deregulation of genes enriched in biological processes that were rather dissimilar to those observed after treatment with *P. mirabilis* OMVs or OMV-RNA (Supplementary Figure 7). We further investigated the expression of specific immune response-related genes following incubation or transfection with *P. mirabilis* OMVs or OMV-RNA compared with LPS. Notably, genes such as *CCL20*, *CXCL8*, and *CXCL10* were strongly upregulated after incubation with OMVs for 10 h or 24 h. After incubation with LPS from *P. mirabilis*, the expression of these genes remained mostly unchanged or did not show the same strong upregulation (Supplementary Table 5). Additionally, transfection of Caco-2 cells with OMV-RNA led to stronger changes in the expression of genes like *CX3CL1, IL1B,* or *TLR3*, compared to cells transfected with LPS from *P. mirabilis* (Supplementary Table 5). In summary, our results indicate that *P. mirabilis* OMVs and OMV-RNA can cause transcriptional changes in the Caco­2 cells that are largely independent from the effects caused by LPS alone.

### Bacteria show interaction with Caco-2 derived EVs and altered bacterial growth

**Figure 4.**
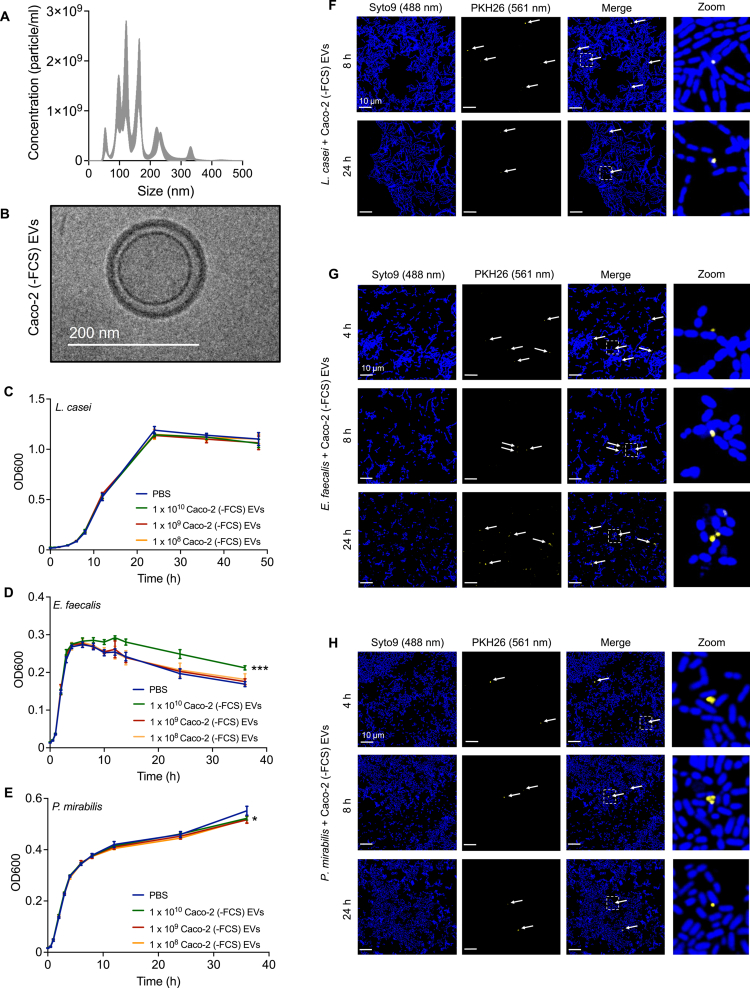
Interaction of *L. casei*, *E. faecalis* and *P. mirabilis* with Caco-2 EVs. (A and B) Characterization of Caco-2 EVs. (A) Typical size distribution of purified Caco-2 (-FCS) EVs measured by NTA. The size distribution is presented as mean ± standard error. (B) Representative cryo-TEM images of Caco-2 (-FCS) EVs showed round shaped particles. (C–E) Growth of *L. casei* (C), *E. faecalis* (D), and *P. mirabilis* (E) in the presence of different amounts of Caco-2 (-FCS) EVs or PBS as a control over a period of 48 h or 36 h at 37 °C. Results are presented as mean ± SD from 4 independent biological replicates. (F–H) Visualization of the interaction of *L. casei* (F), *E. faecalis* (G) or *P. mirabilis* (H) with Caco-2 (-FCS) EVs. Bacteria were incubated for 4 h, 8 h or 24 h with PKH26-labeled Caco-2 (-FCS) EVs. After incubation, bacteria were stained with SYTO™ 9 and fixed. SYTO™ 9 was visualized using a laser at 488 nm, and PKH26 with a laser at 561 nm. For better illustration, the bacteria are shown in blue, and EVs are displayed in yellow. The white arrows indicate areas where PKH26-labeled Caco-2 (-FCS) EVs were detected. Scale bar: 10 µm. Statistical significance was determined using a two-tailed unpaired t-test (*p* < 0.05*, *p* < 0.001***).

In the following, we examined whether bacteria can interact with Caco-2-derived EVs and how this affects their growth. To prevent influences by FCS-derived EVs, we used EVs from Caco-2 cells that were cultured in FCS-free medium. The isolated Caco-2 (-FCS) EVs had a mode size of 121.2 (±6.1) nm and a concentration of 1.2 × 10^11^ (±2.7 × 10^10^) particles/ml (Figure 4A and B). Notably, we observed that *E. faecalis* cultivated in the presence of 1 × 10^10^ Caco-2 EV displayed a prolonged stationary phase, as inferred from OD measurements, prior to entering death phase. In contrast, the growth of *L. casei* remained unaffected when cultivated in the presence of different amounts of Caco-2 EVs. Likewise, we initially detected no effect of the EVs on the growth for *P. mirabilis*. However, after 36 h, bacterial growth was notably reduced in cultures cultivated with Caco-2 EVs (Figure 4C–E). To further explore the interaction of bacteria with eukaryotic EVs, we cultivated them with PKH26-labeled Caco-2 EVs and assessed the localization using CLSM. For *L. casei* and *P. mirabilis*, only weak fluorescence signals for the labeled EVs were detected in proximity to the bacteria. In contrast, for *E. faecalis*, we observed a more frequent association of labeled EVs in the vicinity or co-localizing with the bacterial cells (Figure 4F–H).

### EVs from conditioned and unconditioned media exhibit distinct miRNA expression profiles

**Figure 5.**
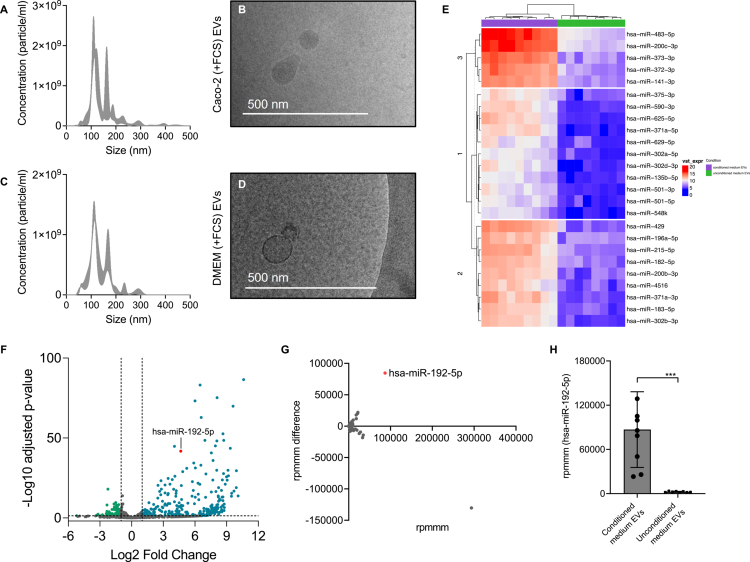
miRNA expression profiles of conditioned and unconditioned medium EVs. (A–D) Characterization of Caco-2 EVs and DMEM EVs. Typical size distribution of purified Caco-2 (+FCS) EVs (A) and DMEM (+FCS) EVs (C) measured by NTA. The size distribution is presented as mean ± standard error. Representative cryo-TEM images of Caco-2 (+FCS) EVs (B) and DMEM (+FCS) EVs (D) showed round-shaped particles. (E–H) Analysis of smallRNA seq data. (E) Hierarchically clustered heatmap of top 25 miRNAs with the highest variance. (F) Scatter plot of differentially expressed miRNAs. The x-axis shows the Log2 fold change, and the y-axis shows the -Log10 of the Benjamini-Hochberg-adjusted *p*-value. Each dot represents a single miRNA. Displayed in green are miRNAs whose expression is significantly (*p* < 0.05) and at least two-fold reduced (Log2 fold change < –1) in the EVs from the conditioned medium. Displayed in blue are miRNAs whose expression is significantly (*p* < 0.05) and at least two-fold increased (Log2 fold change > 1) in the EVs from the conditioned medium. MiR-192-5p is displayed in red. (G) Scatter plot of the miRNA abundance in EVs from conditioned and unconditioned medium. The x-axis shows the rpmmm, and the y-axis shows the rpmmm difference. Each dot represents a single miRNA. An rpmmm difference < 0 indicates a lower abundance, an rpmmm difference > 0 indicates a higher abundance of the respective miRNA in EVs from the conditioned medium. MiR-192-5p is displayed in red. (H) The rpmmm of miR-192-5p in EVs from conditioned and unconditioned medium. Results are presented as mean ± SD from 8-9 independent biological replicates. Statistical significance was determined using a two-tailed unpaired t-test (*p* < 0.001***).

Moreover, we investigated which miRNAs are released from Caco-2 cells within EVs. To distinguish between miRNAs derived from Caco-2 EVs and FCS-EV, we first isolated EVs from Caco-2 cells cultured in FCS-containing medium and, in parallel, from the same FCS-containing medium itself. Caco-2 (+FCS) EVs had a mode size of 123.1 (±10.4) nm and a concentration of 1.1 × 10^11^ (±2.8 × 10^10^) particles/ml. DMEM (+FCS) EVs displayed a similar size of 117.4 (±6.9) nm and concentration of 7.4 × 10^10^ (±1.3 × 10^10^) particles/ml (Figure 5A–D). By using western blot analysis, we confirmed the presence of EV marker and absence of cellular marker (Supplementary Figure 8).

Using smallRNA-seq, we then sequenced miRNAs isolated from Caco-2 (+FCS) EVs (conditioned medium) and compared it to the miRNA expression profile of DMEM (+FCS) EVs (unconditioned medium). Performing hierarchical clustering, we found a discernible clustering pattern based on whether EVs were isolated from conditioned or unconditioned medium (Figure 5E). Differential expression analysis identified a total of 716 miRNAs in EVs from both conditions (Supplementary Table 6). Notably, 252 miRNAs were significantly upregulated (Log2 FC > 1; *p* < 0.05) in EVs from conditioned medium, suggesting that these miRNAs are likely secreted by Caco-2 cells. Conversely, 74 miRNAs were significantly downregulated (Log2 FC < –1; *p* < 0.05) in EVs from conditioned compared with unconditioned medium, making an origin from Caco-2 cells highly unlikely (Figure 5F). To further identify miRNAs that are most likely secreted in EVs by Caco-2 cells, we determined how often a given miRNA was read in relation to 1,000,000 reads (rpmmm, reads per mapped million miRNAs). Among these miRNAs, we identified miR-192-5p, which displayed a 36.4-fold increased abundance in the EVs from conditioned medium, with an rpmmm of 86,918.6 (±51,338.3) compared to an rpmmm of 2,385.1 (±702.0) in EVs from unconditioned medium (Figure 5G and H), suggesting its potential relevance in Caco-2 EV-mediated signaling.

### Bacterial interaction with miR-192-5p is species-dependent and altered by liposomal packaging

**Figure 6.**
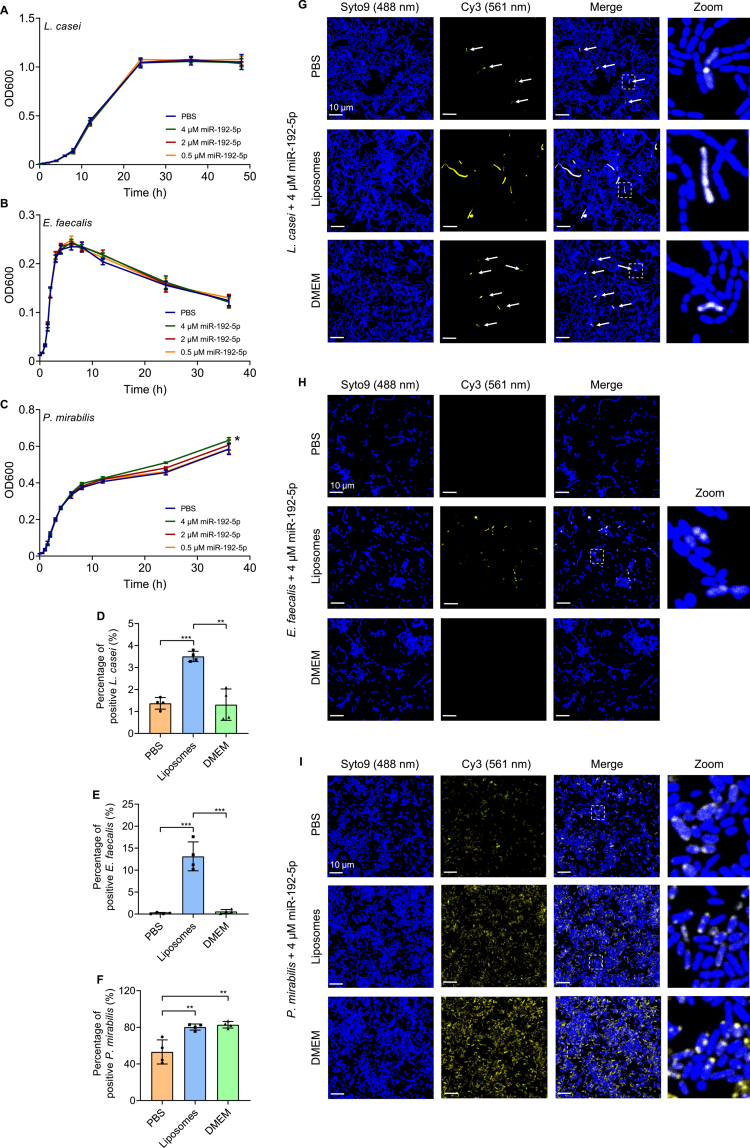
Interaction of *L. casei*, *E. faecalis* and *P. mirabilis* with free and liposome-packaged miR-192-5p. (A–C) Growth of *L. casei* (A), *E. faecalis* (B), and *P. mirabilis* (C) in the presence different concentrations of synthetic miR-192-5p or PBS as a control over a period of 48 h or 36 h at 37 °C. Results are presented as mean ± SD from 3-4 independent biological replicates. (D–F) Flow cytometry analysis of bacteria. *L. casei* (D), *E. faecalis* (E), and *P. mirabilis* (F) were cultivated in the presence of 4 μM free Cy3-labeled synthetic miR-192-5p in PBS or DMEM, or 4 μM liposome-packaged Cy3-labeled synthetic miR-192-5p for 24 h (*E. faecalis* or *P. mirabilis*) or 48 h (*L. casei*) at 37 °C. After the incubation, bacteria were stained with SYTO™ 9 and fixed. Shown is the percentage of SYTO™ 9/Cy3-positive bacteria. Results are presented as mean ± SD from 4 independent biological replicates. (G–I) Visualization of the interaction of *L. casei* (G), *E. faecalis* (H) or *P. mirabilis* (I) with free or liposome-packaged fluorescently labeled synthetic miRNA. Bacteria were cultivated in the presence of 4 μM free Cy3-labeled synthetic miR-192-5p in PBS or DMEM, or 4 μM liposome-packaged Cy3-labeled synthetic miR-192-5p for 24 h (*E. faecalis* or *P. mirabilis*) or 48 h (*L. casei*) at 37 °C. After incubation, bacteria were stained with SYTO™ 9 and fixed. SYTO™ 9 was visualized using a laser at 488 nm, and Cy3 with a laser at 561 nm. For better illustration, the bacteria are shown in blue, and synthetic miRNA is displayed in yellow. The white arrows indicate areas where weak fluorescence signals for Cy3-labeled synthetic miR-192-5p were detected. Scale bar: 10 µm. Statistical significance was determined using a two-tailed unpaired t-test (*p* < 0.05*, *p* < 0.01**, *p* < 0.001***).

Following the identification of miR-192-5p as one of the most abundant miRNAs detected in EVs derived from Caco-2 cells, we hypothesized that this miRNA might exert functional effects on bacterial cells. To test whether miR-192-5p influences bacterial growth, we cultivated them in the presence of increasing concentrations of synthetic miR-192-5p. While no significant growth changes were observed in *L. casei* and *E. faecalis*, we found that exposure to 4 µM synthetic miR-192-5p significantly enhanced (two-tailed unpaired t-test, *p* < 0.05) the growth of *P. mirabilis*, as inferred from OD measurements (Figure 6A–C). To investigate whether the growth enhancement was due to an increased number of viable bacteria, we assessed bacterial viability post-incubation. However, the viability of the bacteria remained unchanged across all three tested species after incubation with 4 µM synthetic miRNA. This suggested that the increased optical density of *P. mirabilis* cultures was rather due to factors other than increased bacterial viability (Supplementary Figure 9A–C).

We next examined whether the bacteria showed an association with miR-192-5p. Therefore, bacterial cultures were cultivated with 4 µM Cy3-labeled synthetic miRNA and analyzed via flow cytometry and CLSM. While only minimal fluorescence signals were detected for *L. casei* and *E. faecalis*, 53.1 (±13.2) % of *P. mirabilis* showed a positive fluorescent signal and a visible accumulation of labeled synthetic miR-192-5p in the proximity of the bacterial cells (Figure 6D–I). This observation indicates species-specific differences in the interaction of bacteria with miR-192-5p.

Since we found that miR-192-5p is present in EVs released from Caco-2 cells, we further investigated whether the physical presentation of miRNAs influences their association with bacterial cells. To address this question, we used liposomes (Lipofectamine™ 3000-based lipid complexes) as a technical delivery system and cultivated the bacteria in the presence of 4 µM liposome-packaged synthetic miRNA. First, we assessed potential effects on bacterial viability. Interestingly, *L. casei* showed a mild decrease in viability following incubation with empty liposomes, which was not observed when the bacteria were incubated with synthetic miRNA-containing liposome. To determine whether this response was specific to miR-192-5p, we used polyadenylic acid [poly(A)] as an alternative nucleic acid cargo. We observed that poly(A)-containing liposomes similarly reduced the viability of *L. casei*. In *E. faecalis*, viability remained unaffected after incubation with any of the liposomes. In contrast, we observed increased viability of *P. mirabilis* in response to incubation with all liposome treatments, including empty liposomes, synthetic miR-192-5p containing liposomes, and poly(A)-containing liposomes (Supplementary Figure 9D–F), indicating a cargo-independent response.

We then evaluated whether liposomal packaging of miRNAs altered the detectability of bacterial interaction with miR-192-5p. Upon incubation of bacteria with 4 µM liposome-packaged Cy3-labeled synthetic miR-192-5p, we detected fluorescent signals from the labeled miRNA in 3.5 (±0.2) % of *L. casei*, 13.1 (±3.3) % of *E. faecalis*, and 80.3 (±3.4) % of *P. mirabilis* (Figure 6D–F). In accordance with these results, we observed an increased accumulation of liposome-packaged fluorescently labeled synthetic miR-192-5p in the proximity of bacteria using CLSM (Figure 6G–I). Collectively, these results indicate that liposomal packaging altered the detectable interaction of bacterial cells with synthetic miR-192-5p.

To further elucidate whether the miRNA packaging impacts the association with the bacteria, we investigated the impact of the incubation media. For liposome-based experiments, bacteria were cultivated in a mixture of their respective growth medium and DMEM, as the latter was used for lipid-complex preparation. In contrast, incubation with the free miRNA was performed in growth medium together with PBS. To exclude the possibility that DMEM itself had an influence on the association of the bacteria with the liposome-packaged miRNA, we repeated the analyzes using free miRNA in DMEM. In *L. casei* and *E. faecalis*, we detected comparable levels of bacteria showing a positive fluorescence signal for the Cy3-labeled synthetic miR-192-5p to those observed in PBS, indicating minimal influence of the DMEM. Notably, also 82.8 (±3.6) % of *P. mirabilis* exhibited fluorescent signals after incubation with the free labeled miRNA in DMEM, with a strong accumulation of these signals in close proximity to the bacteria (Figure 6D–I). This suggests that DMEM itself may modulate the interaction of *P. mirabilis* with miR-192-5p. In summary, our results demonstrate that lipid-based packaging of miR-192-5p modulates its detectable association with the bacteria.

## Discussion

Cross-kingdom communication via EVs represents an emerging mechanism through which microbial and host cells exchange molecular information.^2^ While previous studies have described EVs in cross-kingdom contexts, our work integrates these observations into a unified, bidirectional framework. We propose that EVs and their RNA cargo function not merely as passive transporters, but as directionally active messengers that selectively modulate gene expression and growth behavior in recipient cells across kingdom boundaries.

We observed a time-dependent interaction of Caco-2 cells with BEVs derived from *L. casei*, *E. faecalis* and *P. mirabilis* with species-specific differences in the extent of detectable BEV-associated fluorescence in the cells. While flow cytometry and CLSM cannot definitely distinguish between surface-bound and internalized BEVs, the observed localization patterns are consistent with previous descriptions of BEV-host cell interactions and putative uptake.^34^^,^^39^^,^^60^ Differences observed between the bacterial species may be influenced by structural components of the bacterial envelope, such as cell wall polysaccharides in *E. faecalis*, which have been found to modulate host-microbe interactions,^61^ or LPS in the outer membrane of Gram-negative bacteria like *P. mirabilis*, which stimulates Toll-like receptor 4 (TLR4) on the host cell plasma membrane.^62^ We further observed differences in the amount of RNA associated with the BEVs. Specifically, *P. mirabilis* OMVs exhibited lower RNA levels compared to *L. casei* MVs and *E. faecalis* MVs. *P. mirabilis* OMVs were mostly associated with small RNAs (<200 nts), whereas *L. casei* MVs and *E. faecalis* MVs also carried larger RNA fragments. These results align with other studies showing that RNA cargo of BEVs varies among bacterial species.^8-11^

Using RNA-seq, we demonstrated that BEVs induce distinct transcriptional changes in Caco-2 cells, affecting several thousand of genes. Differentially expressed genes were involved in pathways related to immune signaling, including inflammatory response and chemotaxis, indicating a broad response of the Caco-2 cells to the BEVs. Remarkably, we found that transfection of Caco-2 cells with BEV-RNA alone also results in transcriptional changes, providing mechanistic evidence that RNA cargo contributes independently to the cellular response. These observations align with previous studies reporting that BEV-RNA can modulate host cellular responses.^11^^,^^15^^,^^16^ When analyzing the expression of specific genes, we observed deregulation of genes such as *NOD2*, *NLRP1*, *TLR3*, and *TLR7*, which are involved in microbial and nucleic acid sensing pathways.^63^^,^^64^ Additionally, the altered expression of genes encoding chemokines and interleukins such as *CCL20*, *CXCL8* (*IL-8*), *CXCL10*, *CXCL11*, or *IL-6* suggests the activation of immune signaling pathways.^65^ Changes in the expression of *TLR7*, *CXCL8*, and *CXCL10* upon incubation of Caco-2 cells with MVs from *Listeria monocytogenes* have already been described.^13^ Furthermore, specific RNAs in *Helicobacter pylori* OMVs or *Pseudomonas aeruginosa* OMVs have been shown to modulate the LPS- or OMV-induced secretion of IL-8 in airway epithelial cells or gastric cancer cells.^15^^,^^16^ At the same time, we also observed marked differences in the transcriptomic changes caused by BEVs or BEV-derived RNA alone. Since RNA represents only one of several bioactive components within the BEVs, it is possible that proteins or lipids, which are associated with the BEVs,^3^ may also cause transcriptional changes in recipient cells. OMVs typically contain LPS,^59^ and it has also been reported that LPS can co-purify during RNA isolation from OMV.^66^ Since LPS induces the expression of proinflammatory genes,^62^ we investigated whether the transcriptomic changes in the Caco-2 cells observed upon incubation with *P. mirabilis* OMVs or transfection with the OMV-RNA might be due to the presence of LPS. However, the effects of the OMVs and OMV-RNA differed from those caused by *P. mirabilis* LPS alone, suggesting a distinct role for OMVs and their RNA content in regulating gene expression in host cells. In contrast, another study reported that RNA isolated from *Escherichia coli* OMVs and LPS induce similar gene expression changes in bladder cells.^67^

Conversely, we examined the interaction between EVs derived from Caco-2 cells and different bacterial species. Using CLSM, we demonstrated that fluorescently labeled EVs can be observed in close proximity to *E. faecalis* and that cultivation in the presence of these EVs altered the bacterial growth. Similarly, it has been shown that milk-derived EVs can alter the growth of *Escherichia coli* and *Lactobacillus plantarum*.^68^ However, although, the altered growth of *E. faecalis* might be due to the association of the bacteria with the Caco-2 EVs, the data do not definitively demonstrate a direct causal relationship between the EV association and growth effects. These findings should therefore be interpreted with caution.

The use of EVs derived from cell culture media is a widely used strategy for studying the complex functions of EVs in cell-to-cell communication.^69^ However, a major challenge regarding the analysis of EVs and especially EV-miRNAs from cell culture is the contamination with FCS-derived EVs.^70^ To date, several approaches have been proposed to address this issue, including the use of serum-free media or ultracentrifugation of FCS prior to use.^71^ Nevertheless, serum depletion can impair growth and viability of the cells,^72^ and could potentially alter the EV-miRNA cargo.^73^ To overcome these limitations, we opted for a different approach to identify miRNAs that are secreted within EVs from Caco-2 cells, which avoids the depletion of FCS from the cell culture system by using EVs derived from unconditioned medium as a background control. SmallRNA seq revealed that miR-192-5p is highly abundant in EVs derived from conditioned medium EVs compared with EVs from unconditioned medium, suggesting that this miRNA was released from Caco-2 cells. Consistent with this suggestion, miR-192-5p has been reported to be highly expressed in bowel.^74^

The uptake of mammalian miRNAs by bacteria has thus far been reported in only a limited number of studies.^20-22^^,^^24^ In our study, we observed species-specific differences in the detectable association of bacteria with miR-192-5p, with *P. mirabilis* exhibiting the highest level. CLSM revealed that miR-192-5p mostly accumulates at the poles of *P. mirabilis*. Given that pili can be localized at bacterial poles,^75^^,^^76^ this association may be mediated by type IV pili, which can be used by some bacteria to take up extracellular DNA.^77^ In contrast, the cell wall of Gram-positive bacteria such as *L. casei* and *E. faecalis* consists of a thick peptidoglycan layer, as well as teichoic acids, cell wall polysaccharides, and proteins.^78^ Additionally, *E. faecalis* possesses DNA defense mechanisms, making the interaction or an uptake of foreign nucleic acids even more difficult.^79^ To explore whether the physical presentation of the miRNA influences the interaction of bacteria with miRNAs, we used liposomes as a technical tool to package miR-192-5p. Notably, lipid-based packaging altered the extent of miRNA association, particularly in Gram-positive bacteria *L. casei* and *E. faecalis*. This observation aligns with a recent finding showing that lipid-polymer hybrid nanoparticles could improve the delivery of ampicillin against intracellular *E. faecalis.*^80^ This suggests that physical presentation of miRNAs in lipid-based structures can modulate the bacterial interaction. However, these findings do not imply definite functional equivalence to EV-mediated communication.

Lastly, we assessed the bacterial viability upon incubation with liposome-packaged miR-192-5p. We did not observe any effects on the viability of *E. faecalis* and a cargo-independent effect on the viability of *P. mirabilis*. In contrast, *L. casei* showed a reduced viability in the presence of empty liposomes but not when incubated with miR-192-5p containing liposomes, suggesting a potential protective effect of the miRNA on bacterial cells. Although it is possible that miRNAs may serve as metabolic substrates for bacteria, the observed effect is not fully consistent with a simple feedstock model, as poly(A)-containing liposomes also lead to a reduced bacterial viability. This suggests that the bacteria response is not only influenced by the presence of the nucleic acids per se, but potentially also by RNA-specific properties.

Several limitations of this study should be acknowledged. As already mentioned, flow cytometry and CLSM cannot unambiguously distinguish between surface-bound and internalized EV or miRNAs. Furthermore, BEV concentrations and BEV-RNA amounts differed between the bacterial species. Accordingly, our experiments were designed to assess whether BEVs and BEV-derived RNA from each species have biological effects per se, rather than to compare the effects of BEVs or BEV-RNA across the different species. In addition, all experiments were conducted under simplified *in vitro* conditions that do not fully recapitulate the intestinal environment, such as anaerobic conditions, the presence of immune cells, or complex microbial communities.

Taken together, our study highlights EVs as bidirectional molecular messengers in host-microbe communication network. These insights not only expand our understanding of host-microbiome interactions but also lay the groundwork for exploring EVs as programmable carriers in targeted therapies. Future studies will be required to analyze the communication in more complex experimental systems, and to study whether these interactions occur *in vivo* or under disease-relevant conditions. Nevertheless, our work introduces a conceptual model in which EVs act as mediators in the molecular dialog between humans and their microbiota.

## Supplementary Material

Supplementary_Table4_final.xlsx Supplementary_Table4_final.xlsx

Supplementary_Figures_final.pdf Supplementary_Figures_final.pdf

Supplementary_Table1_final.xlsx Supplementary_Table1_final.xlsx

Supplementary_Table2_final.xlsx Supplementary_Table2_final.xlsx

Supplementary_Table3_final.xlsx Supplementary_Table3_final.xlsx

Supplementary_Table6_final.xlsx Supplementary_Table6_final.xlsx

Supplementary_Table5_final.xlsx Supplementary_Table5_final.xlsx

Supplementary_Table.docx Supplementary_Table.docx

## Data Availability

RNA-seq and smallRNA-seq data have been deposited at GEO (Accession: GSE297395 and GSE297396) and are publicly available as of the date of publication. Original western blot images have been deposited at Mendeley Data (10.17632/hprgf67zgj.1). Any additional information required to reanalyze the data reported in the paper is available from the lead contacts upon request.
