# Supplementary material for: Extracellular vesicles and their RNA cargo facilitate bidirectional cross-kingdom communication between human and bacterial cells
Source: Gut Microbes. 2026 Feb 20;18(1):2630482. doi: 10.1080/19490976.2026.2630482 (PMC12928640; doi:10.1080/19490976.2026.2630482)
Supplement: Supplementary_Figures_final.pdf [file KGMI_A_2630482_SM3866.pdf]

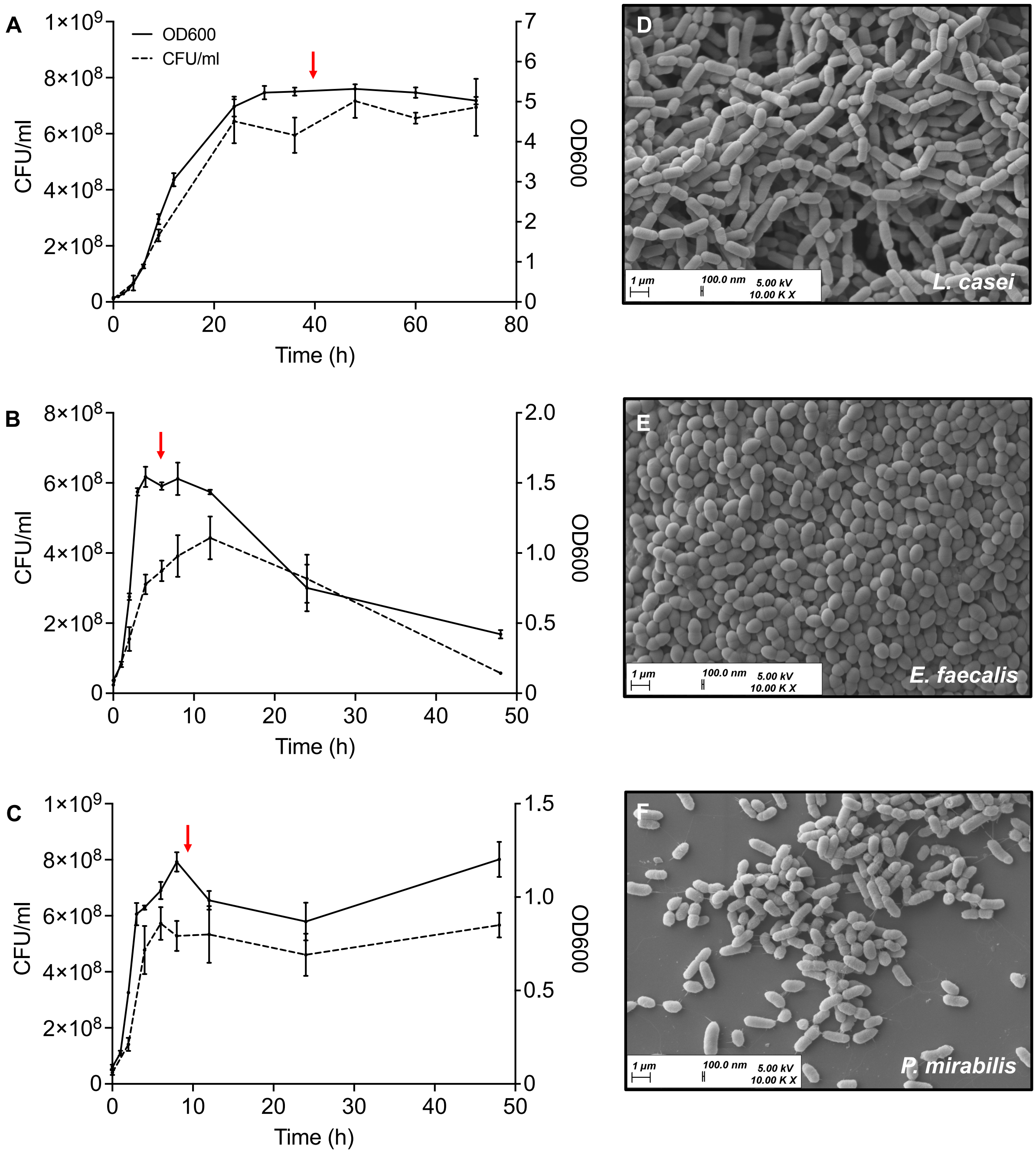

Supplementary Figure 1: Analysis of bacterial growth and morphology.
(**A-C**) Growth of *L. casei* (**A**), *E. faecalis* (**B**), and *P. mirabilis* (**C**) over a period of 72 h or 48 h at 37 °C. Results are presented as mean ± SD from 3-4 independent biological replicates. The red arrows mark the time points where the supernatant for BEV isolation was collected (*L. casei:* 40 h, *E. faecalis:* 6 h, *P. mirabilis:* 9 h). (**D-F**) Scanning electron microscopy images of *L. casei* (**D**), *E. faecalis* (**E**), and *P. mirabilis* (**F**). Bacteria were grown for 40 h (*L. casei*) or 12 h (*E. faecalis* or *P. mirabilis*) at 37 °C. Afterwards, bacteria were fixed, washed, and sputter-coated. Images were taken at 5 kV and 10,000x magnification.

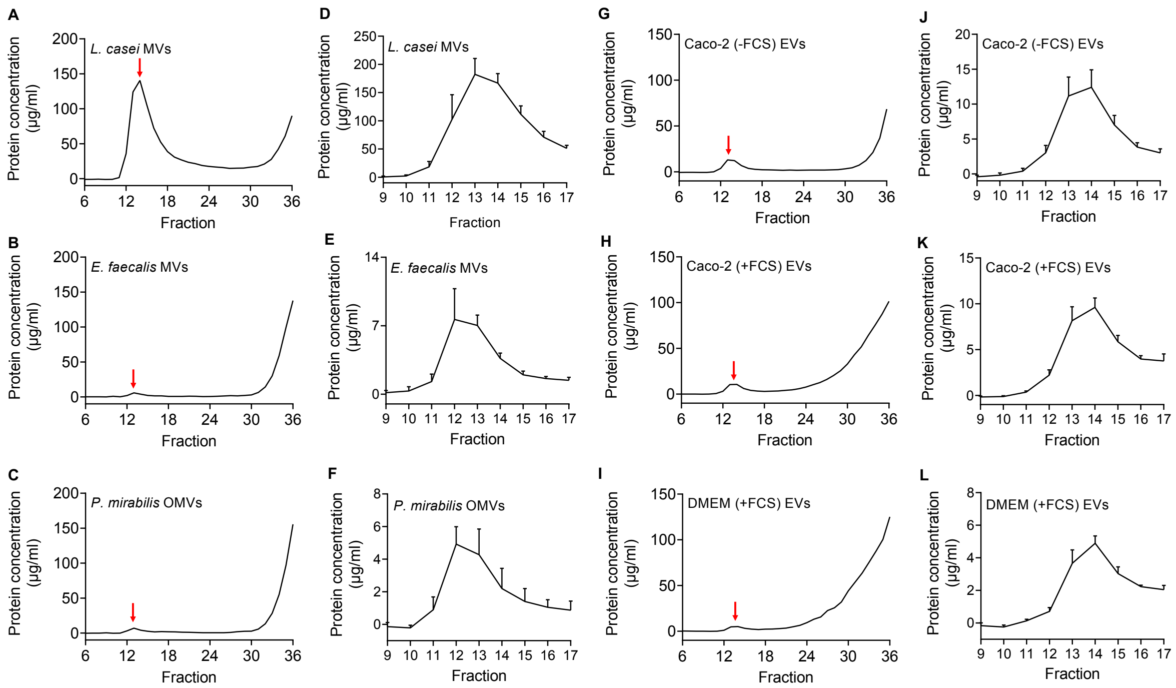

Supplementary Figure 2: Purification of BEVs from *L. casei*, *E. faecalis* and *P. mirabilis* and EVs from different conditions.

(**A-C**) Representative chromatograms after SEC of *L. casei* MVs (**A**), *E. faecalis* MVs (**B**), and *P. mirabilis* OMVs (**C**), demonstrating the successful purification of BEVs. Fractions of 1 ml each were collected. The red arrow marks the fractions where the BEVs can be found. Co-pelleted free proteins and other small contaminants elute in later fractions. (**D-F**) BEVs consistently eluted in fractions 12-15. Results are presented as mean ± SD from 6-8 independent biological replicates, each representing a separate vesicle preparation. (**G-I**) Representative chromatograms after SEC of Caco-2 (-FCS) EVs (**G**), Caco-2 (+FCS) EVs (**H**), and DMEM (+FCS) EVs (**I**), demonstrating the successful purification of EVs. Fractions of 1 ml each were collected. The red arrow marks the fractions where the EVs can be found. Co-pelleted free proteins and other small contaminants elute in later fractions. (**J-L**) EVs consistently eluted in fractions 13-15. Results are presented as mean ± SD from 4 independent biological replicates, each representing a separate vesicle preparation.

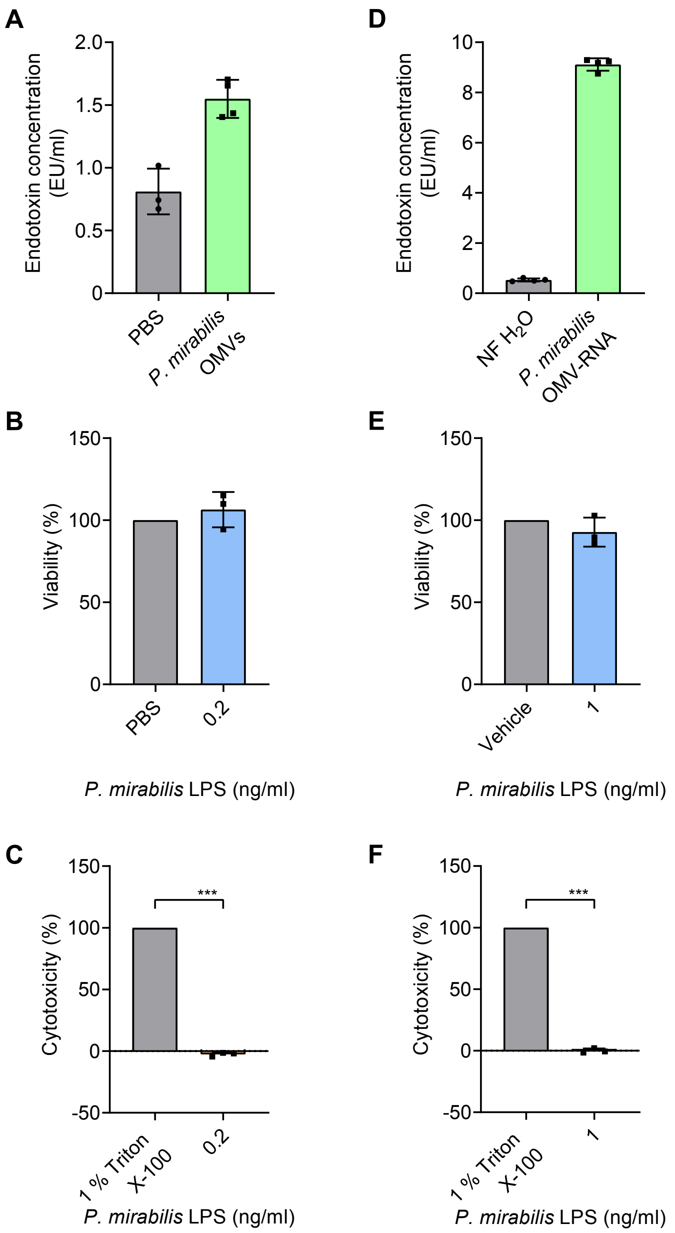

Supplementary Figure 3: Quantification of LPS concentration and effects of LPS on the viability of Caco-2 cells.

(**A-C**) Quantification and effects of LPS in *P. mirabilis* OMVs. (**A**) Endotoxin concentration of purified *P. mirabilis* OMVs in comparison to PBS. (**B and C**) Measurement of cell viability and cytotoxicity after incubation of Caco-2 cells with *P. mirabilis* LPS for 24 h. 1 % Triton X-100 was used as a dead control, whereas PBS was used as a live control. (**D-F**) Quantification and effects of LPS in *P. mirabilis* OMV-RNA. (**D**) Endotoxin concentration of *P. mirabilis* OMV-RNA in comparison to nuclease-free water (NF H_2O). (**E and F**) Measurement of cell viability and cytotoxicity after transfection of Caco-2 cells with *P. mirabilis* LPS for 24 h. 1 % Triton X-100 was used as a dead control, whereas Lipofectamine™ 3000 transfection reagent mixed with nuclease-free water (vehicle) was used as a live control. Results are presented as mean ± standard deviation (SD) from 3-4 independent biological replicates. Statistical significance was determined using a two-tailed unpaired t-test ($p < 0.001$ ***).

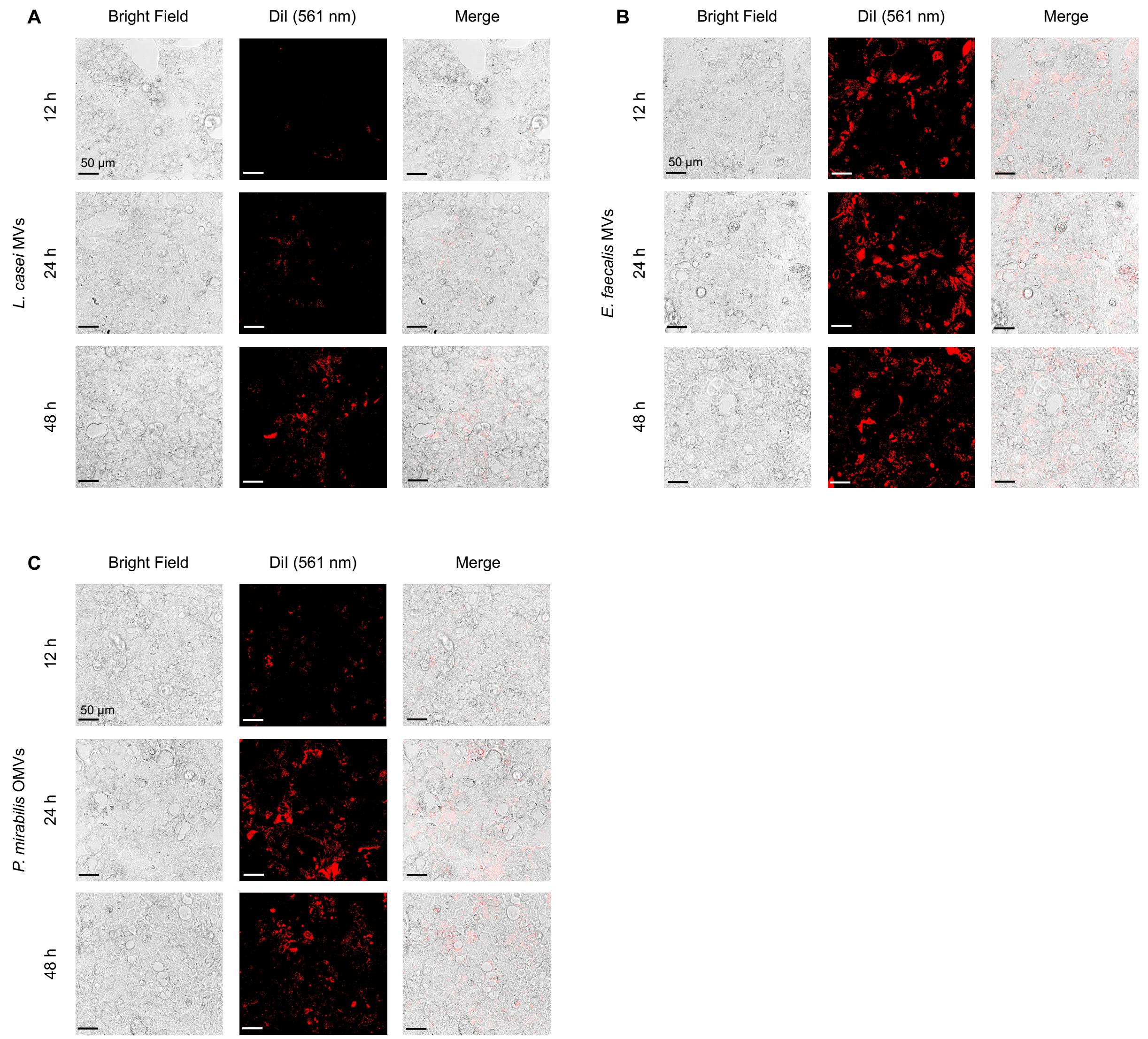

Supplementary Figure 4: Visualization of the interaction of Caco-2 cells with BEVs from *L. casei*, *E. faecalis* and *P. mirabilis*.

(**A-C**) Caco-2 cells were incubated with DiI-labeled *L. casei* MVs (**A**), *E. faecalis* MVs (**B**), or *P. mirabilis* OMVs (**C**) for 12 h, 24 h, or 48 h. After incubation, cells were washed and fixed. Cells were imaged with bright-field, and DiI was visualized using a laser at 561 nm (red). Scale bar: 50 µm.

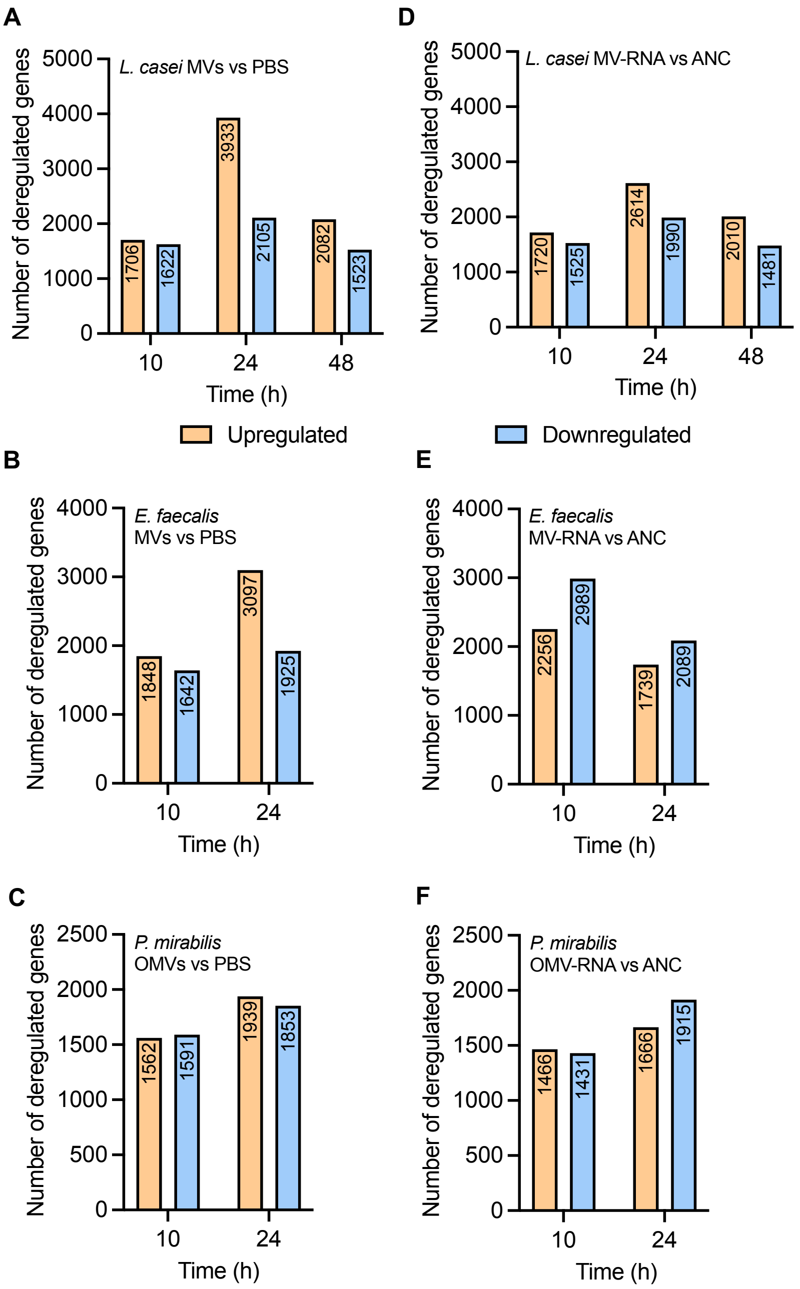

Supplementary Figure 5: Number of deregulated genes after incubation of Caco-2 cells with BEVs or transfection with BEV-RNA.

(**A-F**) Number of deregulated genes after incubation of Caco-2 cells with 9.4×10^5 *L. casei* MVs/cell (**A**), 4.7×10^4 *E. faecalis* MVs/cell (**B**), or 3.75×10^4 *P. mirabilis* OMVs/cell (**C**), or after transfection of Caco-2 cells with 100 ng *L. casei* MV-RNA (**D**), 5 ng *E. faecalis* MV-RNA (**E**), or 2 ng *P. mirabilis* OMV-RNA (**F**) for 10 h, 24 h, or 48 h.

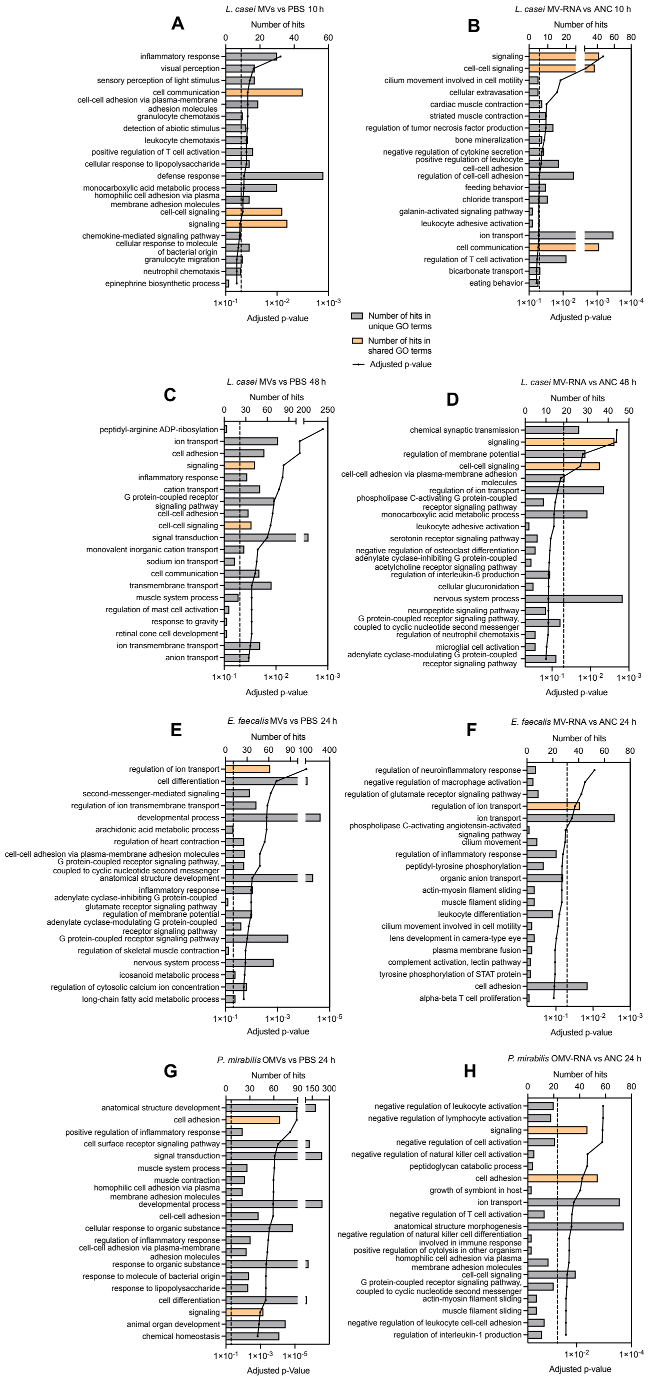

Supplementary Figure 6: Enrichment of genes in specific biological processes after incubation of Caco-2 cells with BEVs or transfection with BEV-RNA.

(**A-H**) Caco-2 cells were incubated with BEVs (*L. casei* MVs: 9.4×10^5 particles/cell, *E. faecalis* MVs: 4.7×10^4 particles/cell, *P. mirabilis* OMVs: 3.75×10^4 particles/cell) or transfected with BEV-RNA (*L. casei* MV-RNA: 100 ng, *E. faecalis* MV-RNA: 5 ng, *P. mirabilis* OMV-RNA: 2 ng) for 10 h, 24 h, or 48 h. Differentially expressed genes were used to perform an over-representation analysis (ORA) using GeneTrail 3.2. Shown are the top 20 Gene Ontology (GO) - biological process in which the differentially expressed genes were enriched in. The dashed line marks the significance level of Benjamini-Hochberg-adjusted p = 0.05. Biological processes that showed an enrichment after incubation with BEVs and transfection of BEV-RNA derived from the same bacteria at the same time point are highlighted in orange.

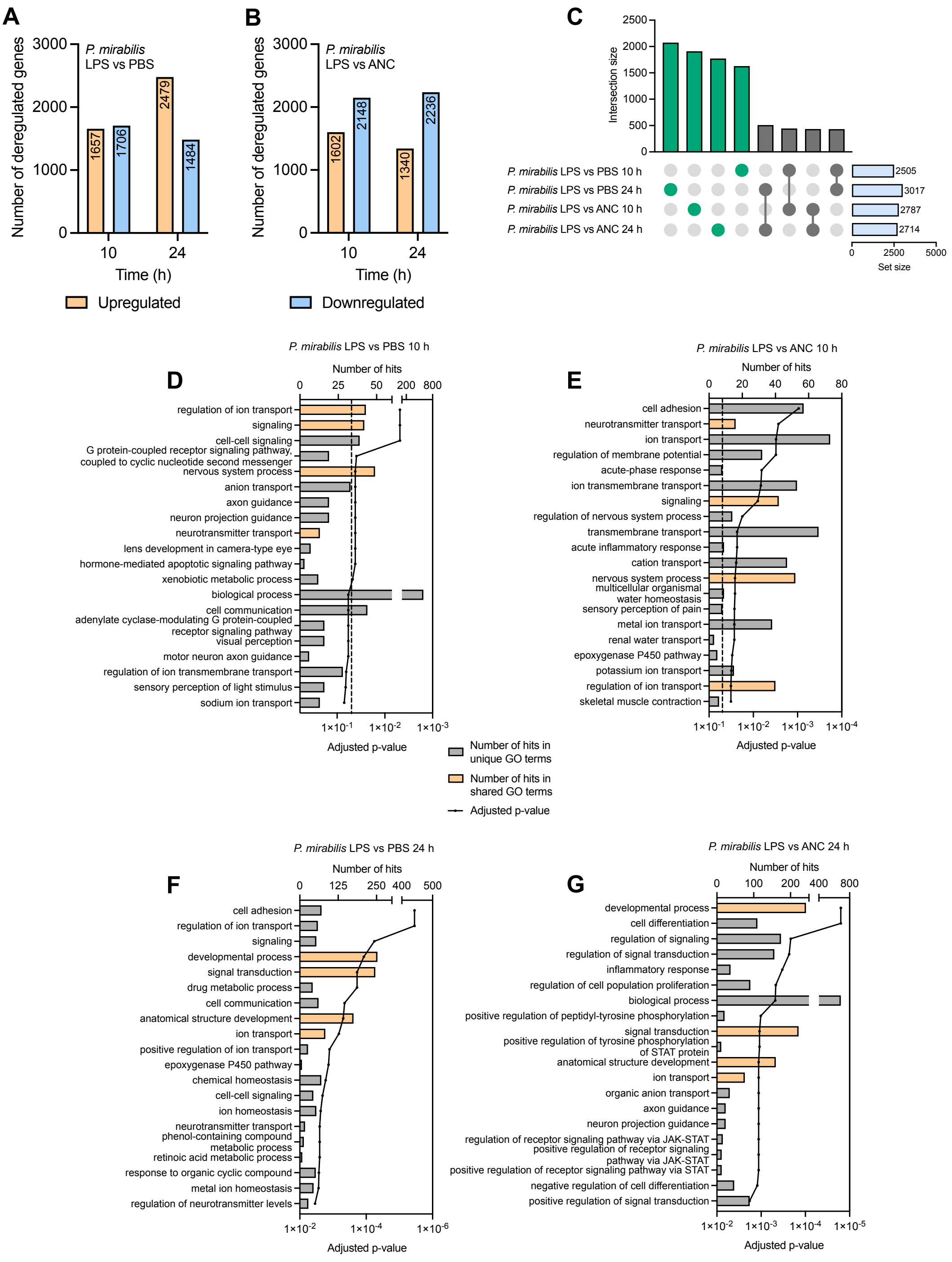

Supplementary Figure 7: Changes in the gene expression of Caco-2 cells after incubation or transfection with *P. mirabilis* LPS.

(**A and B**) Number of deregulated genes after incubation of Caco-2 cells with 100 µl 0.2 ng/ml *P. mirabilis* LPS or transfection with 5 µl 1 ng/ml *P. mirabilis* LPS for 10 h or 24 h. (**C**) Upset plot shows the intersection of differentially expressed genes between the different time points and treatments. (**D-G**) Differentially expressed genes were used to perform an over-representation analysis (ORA) using GeneTrail 3.2. Shown are the top 20 Gene Ontology (GO) - biological process in which the differentially expressed genes were enriched in. The dashed line marks the significance level of Benjamini-Hochberg-adjusted p = 0.05. Biological processes that showed an enrichment after incubation or transfection with *P. mirabilis* LPS at the same time point are highlighted in orange.

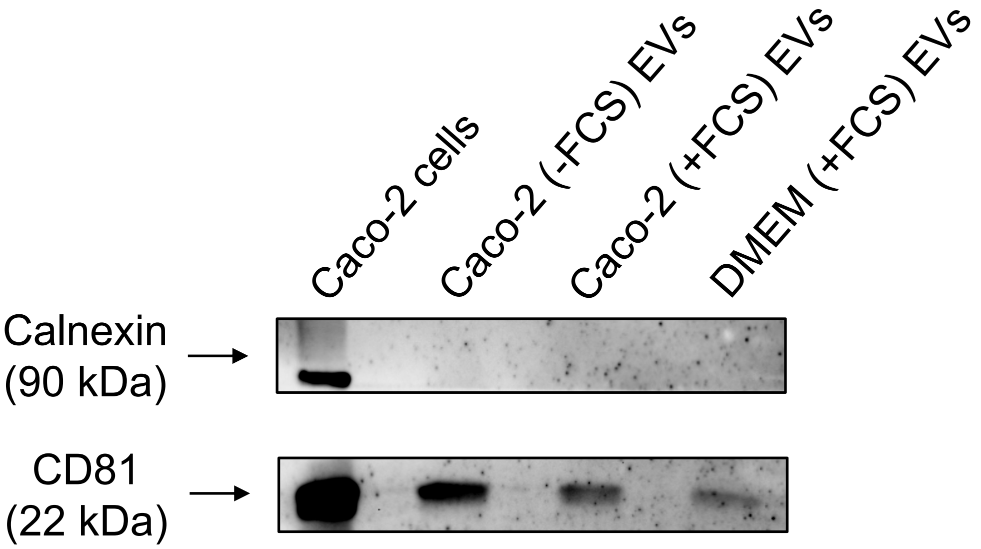

Supplementary Figure 8: Detection of cellular and EV markers using Western Blot analysis. Representative Western blot for calnexin (cellular marker) and CD81 (EV marker) in EVs from different conditions and Caco-2 cells.

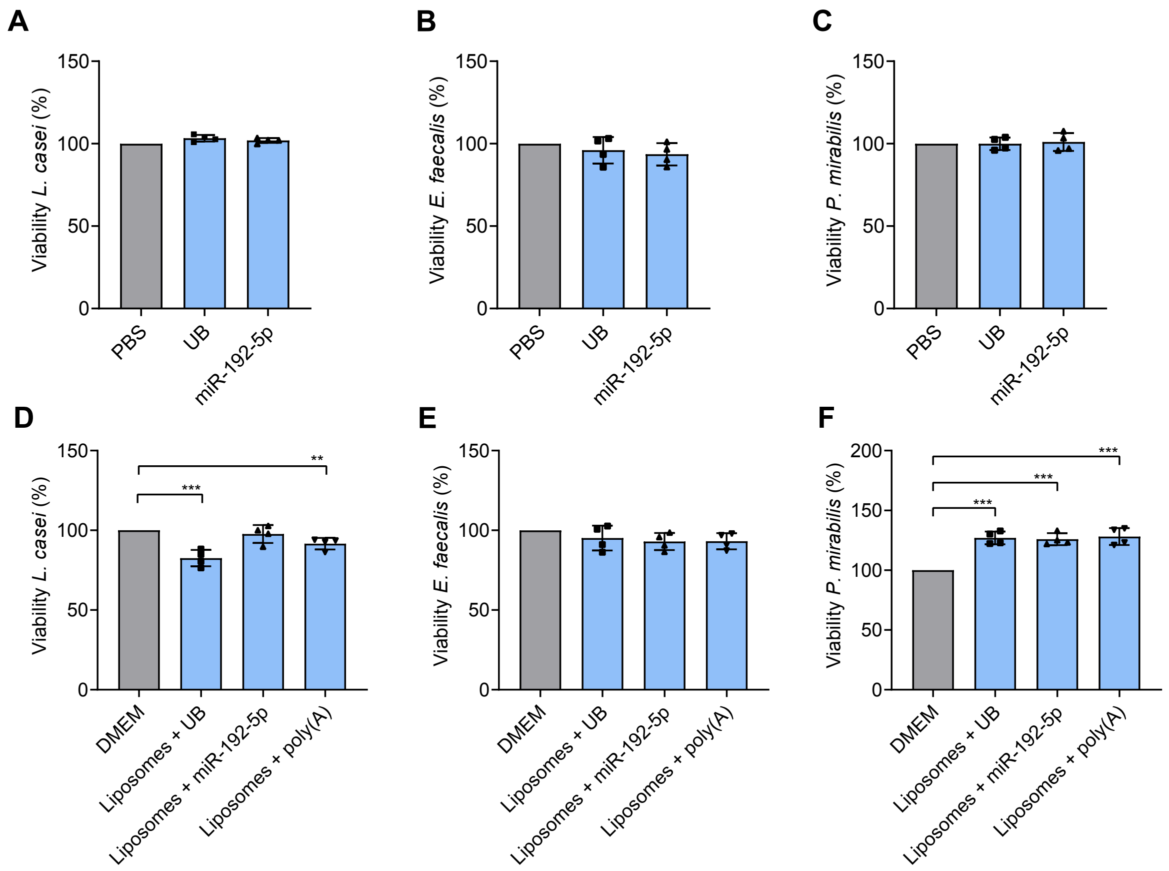

Supplementary Figure 9: Effects of miR-192-5p on the viability of bacteria.

(**A-C**) Viability of bacteria in the presence of free miR-192-5p. Percentage of viable *L. casei* (**A**), *E. faecalis* (**B**) and *P. mirabilis* (**C**) after cultivation in the presence of 4 µM free synthetic miR-192-5p or 6 µl 1 x siMAX Universal Buffer (UB) for 40 h (*L. casei*) or 24 h (*E. faecalis* and P. *mirabilis*) at 37 °C. PBS was used as a control. (**D-F**) Viability of bacteria in the presence of liposome-packaged miR-192-5p. Percentage of viable *L. casei* (**D**), *E. faecalis* (**E**) and *P. mirabilis* (**F**) after cultivation in the presence of 4 µM liposome-packaged synthetic miR-192-5p, liposomes in combination with 6 µl 1 x siMAX Universal Buffer (UB) or 8 µg liposome-packaged poly(A) for 40 h (*L. casei*) or 24 h (*E. faecalis* and P. *mirabilis*) at 37 °C. DMEM was used as a control. Results are presented as mean ± standard deviation (SD) from 4 independent biological replicates. Statistical significance was determined using a two-tailed unpaired t-test ($p < 0.01$ **, $p < 0.001$ ***).
