# Supplementary material for: Extracellular vesicles and their RNA cargo facilitate bidirectional cross-kingdom communication between human and bacterial cells
Source: Gut Microbes. 2026 Feb 20;18(1):2630482. doi: 10.1080/19490976.2026.2630482 (PMC12928640; doi:10.1080/19490976.2026.2630482)
Supplement: Supplementary_Table.docx [file KGMI_A_2630482_SM2230.docx]

**Supplemental information**

Supplementary Table 1. Particle/cell ratios and BEV-RNA amounts used for incubation and transfection.

Supplementary Table 2. RNA sequencing mapping statistics.

Supplementary Table 3. Top 20 enriched GO Biological Processes after incubation or transfection of Caco-2 cells with BEVs or BEV-RNA.

Supplementary Table 4. Expression of selected genes after incubation or transfection of Caco-2 cells with BEVs or BEV-RNA.

Supplementary Table 5. Expression of selected genes after incubation or transfection of Caco-2 cells with *P. mirabilis* OMV, OMV-RNA or LPS.

Supplementary Table 6. Expression of miRNAs in EVs isolated from conditioned and unconditioned medium.
